# Objective functional performance 1 year after total knee arthroplasty does not differ for patients with symptoms of anxiety, depression or pain catastrophizing: A prospective study of 289 patients

**DOI:** 10.1002/jeo2.70645

**Published:** 2026-01-19

**Authors:** Margot B. Aalders, Jelle P. van der List, Stef Daniel, Gino M. M. J. Kerkhoffs, Job N. Doornberg, Ruurd L. Jaarsma, Lucien C. M. Keijser, Joyce L. Benner

**Affiliations:** ^1^ Department of Orthopaedic Surgery and Sports Medicine Amsterdam UMC, Location AMC Amsterdam the Netherlands; ^2^ Centre for Orthopedic Research Alkmaar (CORAL) Alkmaar the Netherlands; ^3^ Department of Orthopaedic Surgery, Flinders Medical Centre Flinders University Adelaide South Australia Australia; ^4^ Department of Orthopaedic Surgery University Medical Centre Groningen Groningen the Netherlands; ^5^ Department of Orthopaedic Surgery and Sports Medicine The Ohio State University Wexner Medical Center Columbus Ohio USA; ^6^ Sports Medicine Research Institute The Ohio State University, Wexner Medical Center Columbus Ohio USA; ^7^ Amsterdam Movement Sciences, Programs Sports and Musculoskeletal Health Amsterdam the Netherlands; ^8^ Academic Center for Evidence‐Based Sports Medicine (ACES), Amsterdam UMC Amsterdam the Netherlands; ^9^ Department of Orthopaedic Surgery NorthWest Clinics Alkmaar the Netherlands

**Keywords:** functional performance, knee arthroplasty, mental health, range of motion, strength, timed up and go

## Abstract

**Purpose:**

Preoperative anxiety, depression and pain catastrophizing (PC) symptoms are associated with inferior patient‐reported outcomes after total knee arthroplasty (TKA). It remains unclear whether such differences also exist for objective outcomes such as strength, range of motion (ROM) and timed up and go (TUG). This study aimed to investigate whether objective functional performance differs up to 12 months postoperatively in patients with these psychological symptoms.

**Methods:**

A prospective cohort of 289 TKA patients was analysed (55% female, age 71 [65–76]). Anxiety (21%), depression (17%) and PC (14%) were assessed preoperatively using the Hospital Anxiety and Depression Scale and Pain Catastrophizing Scale. Objective outcomes consisting of maximum strength (strength), strength endurance (SE), ROM and TUG were measured preoperatively and at 6 and 12 months postoperatively. Between‐group comparisons were adjusted for confounders (age, sex, American Society of Anesthesiologists, body mass index, surgical approach, baseline functional performance) using multivariable regression.

**Results:**

Preoperatively, mainly anxiety‐ and depression‐symptoms were associated with lower strength and SE (all *p* < 0.05), and TUG was worse in depression‐ and PC patients (all *p* ≤ 0.021). ROM did not differ between groups. At 6 and 12 months, unadjusted analyses showed continued associations between anxiety/depression and inferior flexion strength or SE (all *p* ≤ 0.023), but these either resolved by 12 months (*p* = 0.843) or lost significance after multivariable adjustment (*p* ≥ 0.052), except for flexion strength in anxiety patients (*p* = 0.033). PC symptoms were associated with greater TUG improvement at 12 months (*p* = 0.005), though minimal clinically important difference attainment and ROM outcomes remained similar across groups.

**Conclusion:**

Although preoperative psychological symptoms are associated with poorer objective outcomes, these differences resolve by 6–12 months postoperative follow‐up. After 1 year, similar objective outcomes are attained for patients with symptoms of anxiety, depression or PC. Interpreting this in the bigger scope, the importance of integrating psychological support into the perioperative pathway to align subjective and objective outcomes is underlined.

**Level of Evidence:**

Level II, prognostic cohort study.

**Abbreviations**
ASA: American Society of Anesthesiologists
BMI: body mass index
CI: confidence interval
HADS: Hospital Anxiety and Depression Scale
HADS‐A: Hospital Anxiety and Depression Scale—Anxiety Subscale
HADS‐D: Hospital Anxiety and Depression Scale—Depression Subscale
IQR: interquartile range
KOOS: Knee Injury and Osteoarthritis Outcome Score
MCID: Minimal Clinically Important Difference
Nm/kg: Newton‐metre per kilogram bodyweight
OKS: Oxford Knee Score
PASS: Patient Acceptable Symptom Scale
PC: pain catastrophizing
PCS: Pain Catastrophizing Scale
PROMs: patient‐reported outcome measures
ROM: range of motion
SD: standard deviation
SE: strength endurance
STROBE: Strengthening the Reporting of Observational Studies in Epidemiology
TKA: total knee arthroplasty
WOMAC: Western Ontario and McMaster Universities Osteoarthritis Index

## INTRODUCTION

Despite large improvements in postoperative satisfaction following total knee arthroplasty (TKA) over the decades, a considerably high rate (around 10%–15%) of dissatisfied patients persists [10, 32]. Causes of patients' dissatisfaction have been investigated thoroughly and primarily concern residual pain and patient‐reported malfunction [13]. In the last decade, awareness has grown on the importance of a patients' psychological state in the causality of dissatisfaction, visible through the vastly increased publication density on this topic [1, 13, 15, 23, 26]. Accordingly, many studies have shown significant associations between preoperative psychological health (e.g., anxiety, depression and pain catastrophizing [PC]) and inferior subjective postoperative outcomes (patient‐reported outcome measures, PROMs) after TKA [1, 13, 15, 23]. These subjective outcomes often concern function, pain, satisfaction and quality of life PROMs [1, 13, 15, 23]. Thus, the influence of psychological health on subjective outcomes has been investigated thoroughly and is accepted as an important association in the literature [1, 13, 15, 23, 26].

However, the impact on objective functional outcomes, for example, strength, range of motion (ROM) and timed up and go (TUG), remains unclear. Some prior studies assessed the influence of psychological health on objective TKA outcomes such as revision rates or complications, but did not include patient‐centred outcomes [15, 24]. As patients report malfunction, besides the intrinsically subjective complaint of pain, as the most substantial reason for dissatisfaction, an important lack of knowledge exists on the association between psychological health and objectively assessed function [14, 16, 22]. Literature on this association is sparse and has been investigated in only a few studies, though with a limited follow‐up or only ROM as the objective outcome [14, 16].

Therefore, this study aimed to be the first to prospectively investigate whether objective functional outcomes after TKA differ between patients with and without preoperative anxiety, depression and PC symptoms, up to 1‐year follow‐up. It was hypothesized that no differences between these patients would be found in objective outcome measures.

## METHODS

This prospective cohort study was conducted at a high‐volume teaching hospital in the Netherlands from 1 January 2019 to 31 December 2023. Institutional review board approval was obtained (L019‐075/FOLLOW), and all participants provided written informed consent prior to inclusion. Data collection followed STROBE and RECORD guidelines [2, 11].

### Patient population

Patients were eligible for inclusion if they (1) were proficient in Dutch, (2) completed preoperative psychological health questionnaires and objective functional performance measurements, (3) underwent unilateral primary TKA for symptomatic advanced osteoarthritis [21] and (4) provided written informed consent. Exclusion criteria were (1) failure to attend the 1‐year follow‐up functional performance measurements after two rescheduling attempts or (2) withdrawal during follow‐up.

Psychological health status was assessed preoperatively using PROMs, including the Hospital Anxiety and Depression Scale (HADS) [38] and the Pain Catastrophizing Scale (PCS) [8]. The HADS consists of anxiety (HADS‐A) and depression (HADS‐D) subscales, each with seven items (max score 21) (Appendix S1). Scores ≥8 indicated anxiety or depression symptoms [5, 38]. The PCS is a validated questionnaire and comprises 13 items scored on a 5‐point Likert scale (total 0–52) (Appendix S2) [27, 34]. Scores ≥30 were considered clinically relevant PC [34].

Patients were categorized based on the presence or absence of anxiety symptoms (anxiety or non‐anxiety patients, respectively), depression symptoms (depression or non‐depression patients, respectively) and PC symptoms (PC or non‐PC patients, respectively). None of the patients received treatment for the psychological symptoms within the study institution.

**Figure 1:**
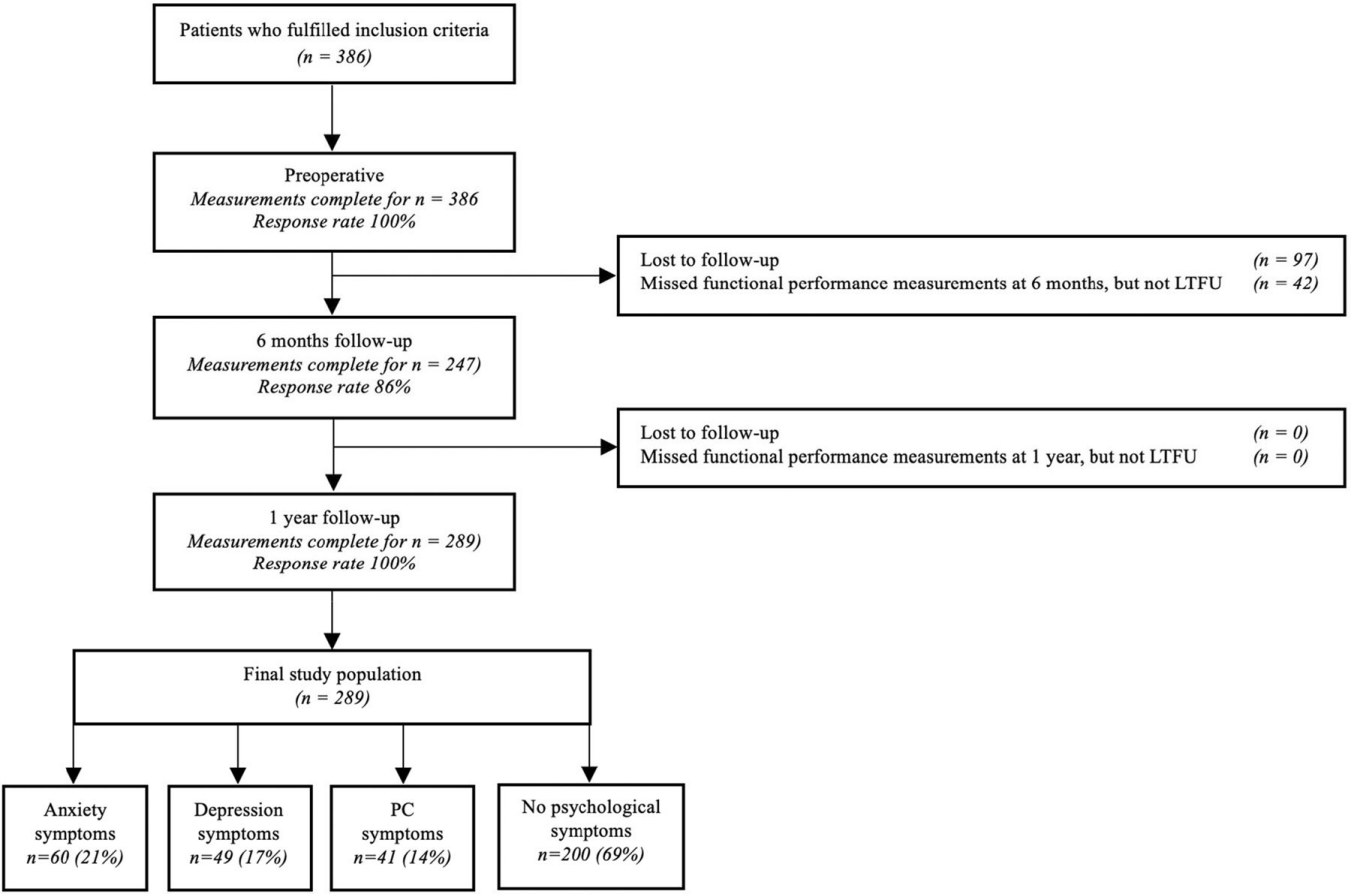
Flow diagram of final study population, including the response rate (percentage of patients that completed the functional performance measurements at that particular follow‐up moment), and number of patients who missed follow‐up functional performance measurements, that is, excluded patients during follow‐up. Exclusion criteria: (1) failure to attend the 1‐year follow‐up functional performance measurements after two rescheduling attempts or (2) withdrawal during follow‐up. LTFU, lost to follow‐up; PC, pain catastrophizing.

**Figure 2:**
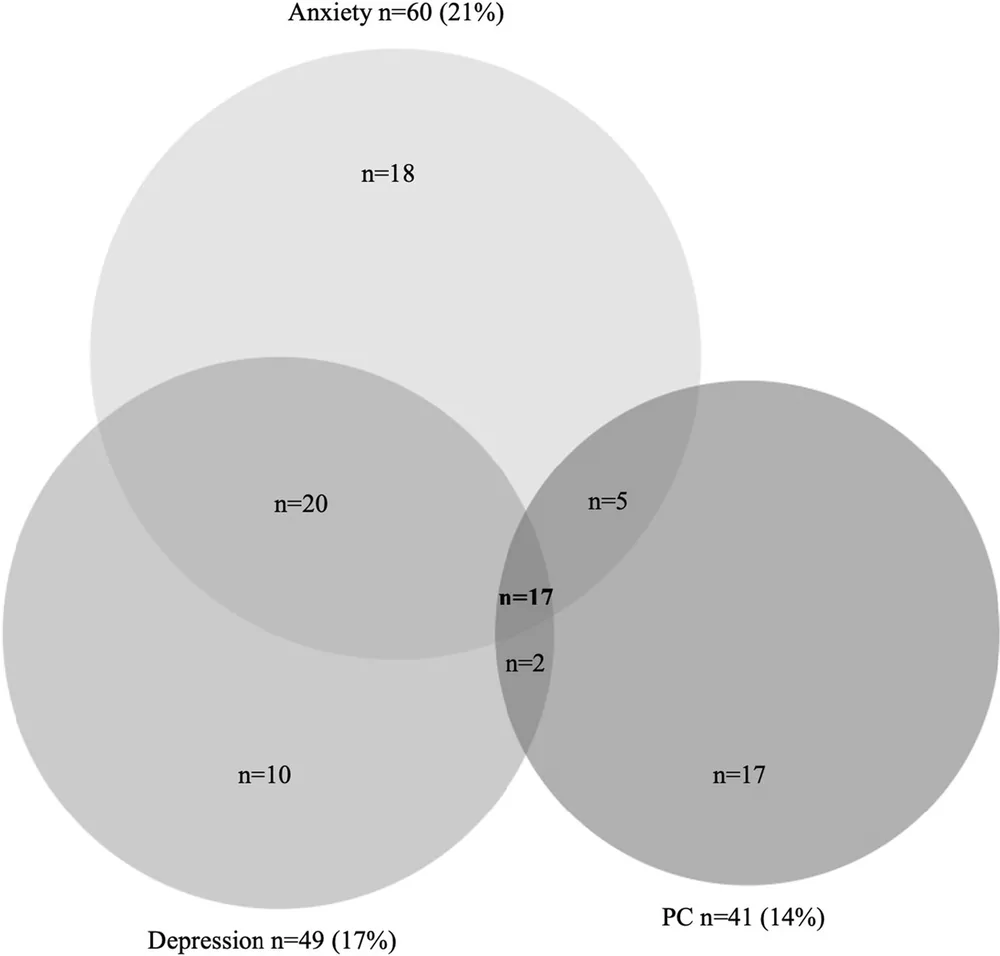
Venn diagram: overlap of preoperative psychological symptoms (*n* = 89) in the study population (*n* = 289), reported in numbers (*n*). The percentages given are based on the total included population (*n* = 289). (C) 2003–2008 Tim Hulsen (BioVenn) [19]. PC, pain catastrophizing.

Within the specified timeframe, 386 patients fulfilled all inclusion criteria, of which 97 withdrew from the study between preoperative assessment and 6 months postoperative follow‐up, resulting in a final cohort of 289 patients (Figure 1). A summary of baseline characteristics and perioperative characteristics is listed in Table 1. Of the 289 patients, 89 patients had preoperative psychological symptoms. Anxiety, depression and PC were present in 60 (21%), 49 (17%) and 41 (14%) patients, respectively, with reasonable overlap (7% of the study population had anxiety and depression symptoms, 2% had anxiety and PC symptoms, 1% had depression and PC symptoms, and 6% had the combination of anxiety, depression and PC symptoms, Figure 2).

**Table 1:** Baseline characteristics and intra‐ and postoperative demographics for all patients who underwent TKA, stratified by anxiety, depression and PC.

	Total population (*n* = 289)	Anxiety (*n* = 60)	Non‐anxiety (*n* = 229)	Depression (*n* = 49)	Non‐depression (*n* = 240)	PC (*n* = 41)	Non‐PC (248)
Baseline variables							
Age (years)^a^	71.0 (65–76)	70.5 (61–75)	71.0 (65–76)^†^	70.0 (61–75)	71.0 (65–76)^†^	68.0 (58–74)	72.0 (65–76)*
Female gender^b^	160 (55.4)	41 (68.3)	119 (52.0)*	31 (63.3)	129 (53.8)^†^	26 (63.4)	134 (54.0)^†^
BMI (kg/m^2^)^c^	28.6 ± 4.3	29.2 ± 4.4	28.5 ± 4.3^†^	29.2 ± 5.4	28.5 ± 4.1^†^	30.5 ± 4.6	28.3 ± 4.2*
Smoking^b^	17 (5.9)	5 (8.3)	12 (5.2)^†^	4 (8.2)	13 (5.4)^†^	1 (2.4)	16 (6.5)^†^
ASA I/II^b^	214 (74.0)	41 (68.3)	173 (75.5)^†^	29 (59.2)	185 (77.1)*	31 (75.6)	183 (73.8)^†^
Mean HADS‐anxiety score^a^	4.0 (2–7)	10.0 (8–12)	3.0 (2–5)**	—	—	—	—
Mean HADS‐depression score^a^	3.0 (2–6)	—	—	10.0 (8–11)	3.0 (1–4)**	—	—
Mean PCS score^a^	11.0 (4–21)	—	—	—	—	35.0 (31–43)	9.0 (3–16)**
Right side^b^	142 (49.1)	33 (55.0)	109 (47.6)^†^	30 (61.2)	112 (46.7)^†^	20 (48.8)	122 (49.2)^†^
Physical therapy pre‐operatively^b^	90 (31.1)	20 (50.0)	70 (43.2)^†^	15 (48.4)	75 (43.9)^†^	14 (46.7)	76 (44.2)^†^
Perioperative variables							
Medial parapatellar approach^b^	265 (91.7)	57 (95.0)	208 (90.8)^†^	45 (91.8)	220 (91.7)^†^	41 (100.0)	224 (90.3)*
Patellar resurfacing^b^	54 (18.7)	10 (16.7)	44 (19.2)^†^	8 (16.3)	46 (19.2)^†^	7 (17.1)	47 (19.0)^†^
Surgery duration (minutes)^a^	72.0 (61–83)	69.0 (59–90)	72.0 (62–81)^†^	69.0 (60–89)	72.0 (62–81)^†^	72.0 (60–84)	72.0 (61–82)^†^
Estimated blood loss (mL)^a^	200.0 (100–300)	200.0 (100–325)	200.0 (100–300)^†^	200.0 (100–300)	200.0 (100–300)^†^	175.0 (100–300)	200.0 (100–300)^†^
Length of stay (days)^a^	1.0 (1–2)	1.0 (1–3)	1.0 (1–2)^†^	1.0 (1–3)	1.0 (1–2)*	1.0 (1–2)	1.0 (1–2)^†^
Length of stay ≥3 days^b^	51 (17.6)	15 (25.4)	36 (15.9)^†^	14 (28.6)	37 (15.6)*	9 (22.5)	42 (17.1)^†^

*Note*: Significant differences between patients with and without psychological symptoms are indicated by * for *p* < 0.05, and ** for *p* < 0.001. Insignificant differences are indicated by † for *p* > 0.05.

Abbreviations: ASA, American Society of Anaesthesiologists physical status classification; BMI, body mass index; HADS, Hospital Anxiety and Depression Scale; PC, pain catastrophizing; PCS, Pain Catastrophizing Scale; TKA, total knee arthroplasty; *n*, number of patients.

^a^ Reported in median with interquartile range (lower quartile–upper quartile), Mann–Whitney *U* test used.

^b^ Reported in number (%), *χ* ^2^ test used.

^c^ Reported in mean ± standard deviation, independent *t* test used.

All patients followed standardized institutional postoperative care: four‐week antithrombotic prophylaxis, physical therapy‐guided mobilization, 50% weight‐bearing for 1–3 weeks and progressive full weight‐bearing. Radiographic evaluation was scheduled at 2 and 12 months.

### Data collection and outcome measures

Primary outcomes were measured preoperatively and at 6 and 12 months postoperatively, including knee extension maximum strength (the peak torque corrected for bodyweight, measured at 60°/s, in this study referred to as ‘strength’), knee extension strength endurance (the peak torque corrected for bodyweight, measured at 180°/s, in this study referred to as ‘SE’), ROM and TUG. The TUG test is a validated and widely used performance‐based measure of functional performance. It records the time (in seconds) required for a patient to rise from a standard chair, walk 3 m, turn, return and sit down again. The TUG has shown excellent reliability and validity across diverse populations and correlates strongly with overall lower limb strength, balance and functional independence [17, 18].

Strength and SE were measured in Newton‐metre corrected for bodyweight (Nm/kg) using the Biodex System 4 Pro instrument [35, 37] and reported as absolute value and as deficit of the affected leg undergoing TKA compared to the unaffected contralateral leg, in percentages (%). This strength deficit is defined as ‘muscle imbalance’ of the affected leg compared to the unaffected leg. Percentages of ≤20% are generally used to indicate no significant impairment for the affected compared to the non‐affected leg [4], which were considered acceptable in this study. ROM in degrees (°) using a goniometer and TUG in seconds (s) using a stopwatch. Measurements were conducted at the outpatient clinic by a small team of trained physiotherapists. Missed appointments were attempted to reschedule twice intermittently before patients were classified as lost to follow‐up.

Clinical improvements (*Δ*) from preoperatively to 6 and 12 months postoperatively were calculated, including the proportion of patients exceeding the minimal clinically important difference (MCID) for TUG (−3.4 s) [12]. MCIDs for other outcomes, and PASS for all primary outcomes, were unavailable in the literature. However, for Strength and SE‐deficits, attainment of muscle imbalance percentages of ≤20% was considered as no significant impairment for the affected leg and reasonable recovery. Baseline patient demographics and intra‐ and postoperative characteristics were collected at the time of surgery.

### Statistical analysis

A priori sample size calculation, based on the primary outcome TUG due to the available literature (Cohen's *d* = 0.495 [16], MCID = 3.4 [12]), *α* = 0.05, power 80% and enrolment ratio 5:1, determined a required sample of 234 (39 patients with depression/anxiety/PC symptoms and 195 without). Continuous and categorical variables were presented as mean with standard deviation (SD) and number with percentage (%), respectively. When normality was violated, median (mn) and interquartile range (IQR) were presented. Independent *t* tests and Mann–Whitney *U* tests compared normally and non‐normally distributed function‐measurements and improvement‐scores between groups (non‐anxiety versus anxiety, non‐depression versus depression, non‐PC versus PC). *χ* ^2^ tests compared categorical variables between groups. Significance of differences was reported using *p* values and effect sizes (Cohen's *d*) with 95% confidence intervals (95% CIs). A *p* < 0.05 indicated statistical significance. Independent associations of anxiety, depression and PC with postoperative functional outcomes were analysed and verified using multivariable regression analysis, adjusting for potential baseline confounders (age, sex, body mass index [BMI], American Society of Anesthesiologists classification, surgical approach and baseline functional performance measurements). Multivariable regression results were reported as *β*, 95% CI and *p* values. Baseline functional performance was included since anxiety/depression/PC patients tend to start at lower performance scores.

## RESULTS

### Preoperative differences for anxiety

Preoperatively, some significant differences were found for patients with anxiety, depression and PC symptoms compared to their counterparts. Anxiety patients showed significant lower extension strength (Table 2 and Appendix S3, 60.7 ± 35.0 vs. 71.1 ± 34.9 Nm/kg, *p* = 0.048, *d* = 0.30 [0.0, 0.6]), lower extension SE (Table 3 and Appendix S3, extension: 43.3 ± 23.6 vs. 53.6 ± 24.5 Nm/kg, *p* = 0.005, *d* = 0.42 [0.1, 0.7]) and higher extension SE‐deficits (Table 3 and Appendix S3, extension: deficit of 28.4 ± 27.6 vs. 20.2 ± 27.0%, *p* = 0.041, *d* = −0.30 [−0.6, −0.0]). However, preoperative values for ROM and TUG were similar between anxiety and non‐anxiety patients (Tables 4 and 5, Appendix S3).

**Table 2:** Objective measurements: Maximum strength (peak torque corrected for bodyweight and measured at 60°/s) of affected leg and the percentage strength‐deficit of affected leg compared to the non‐affected leg during extension (quadriceps), preoperatively, 6 and 12 months postoperatively, and differences 0–6 and 0–12 months, in all patients who underwent TKA and for patients stratified by anxiety, depression and PC.

Outcome variables	Total population (*n* = 289)	Anxiety (*n* = 60)	Non‐anxiety (*n* = 229)	Depression (*n* = 49)	Non‐depression (*n* = 240)	PC (*n* = 41)	Non‐PC (248)
Preoperative							
Strength A leg—extension (Nm/kg)	69.0 ± 35.1	60.7 ± 35.0	71.1 ± 34.9*	60.5 ± 35.4	70.8 ± 34.8^†^	60.2 ± 39.3	70.5 ± 34.2^†^
Deficit of A leg—extension (%)	24.2 ± 37.0	25.4 ± 34.7	24.0 ± 37.6^†^	19.1 ± 52.5	25.3 ± 33.0^†^	26.7 ± 35.0	23.8 ± 37.4^†^
6 months postoperative							
Strength A leg—extension (Nm/kg)	71.7 ± 28.7	70.5 ± 33.5	72.0 ± 27.7^†^	64.6 ± 29.2	72.9 ± 28.6^†^	67.8 ± 34.4	72.2 ± 28.0^†^
Deficit of A leg—extension (%)	23.6 ± 39.8	29.6 ± 22.2	22.2 ± 42.7^†^	31.5 ± 27.2	22.2 ± 41.5^†^	32.7 ± 21.1	22.4 ± 41.5^†^
12 months postoperative							
Strength A leg—extension (Nm/kg)	86.0 ± 31.3	80.2 ± 33.8	87.3 ± 30.7^†^	78.4 ± 32.9	87.3 ± 31.0^†^	78.0 ± 37.2	86.9 ± 30.5^†^
Deficit of A leg—extension (%)	14.5 ± 46.3	14.8 ± 27.6	14.4 ± 49.6^†^	12.7 ± 47.0	14.8 ± 46.3^†^	21.9 ± 26.5	13.6 ± 48.0^†^
Δ Strength A leg extension 0–6 months (Nm/kg)	2.7 ± 28.7	5.4 ± 25.0	2.1 ± 29.4^†^	4.3 ± 31.9	2.4 ± 28.1^†^	2.7 ± 28.3	2.7 ± 28.8^†^
Δ Strength A leg extension 0–12 months (Nm/kg)	15.7 ± 29.3	16.1 ± 27.7	15.7 ± 29.7^†^	17.7 ± 31.4	15.4 ± 29.0^†^	11.5 ± 29.3	16.2 ± 29.4^†^

*Note*: Strength in Nm/kg corrected for bodyweight, deficit in %. All reported in mean (mn) ± standard deviation (SD), independent *t* test used. Forty‐two patients missed outcomes at 6 months postoperatively, and all patients had outcomes preoperatively and at 12 months postoperatively. Significant differences between patients with and without psychological symptoms are indicated by * for *p* < 0.05. Insignificant differences are indicated by † for *p* > 0.05.

Abbreviations: A leg, affected leg; kg, kilograms; Nm, Newton‐metre; PC, pain catastrophizing; TKA, total knee arthroplasty; *Δ*, difference.

**Table 3:** Objective measurements: Strength endurance (peak torque corrected for bodyweight and measured at 180°/s) of affected leg and strength‐deficit of affected leg compared to the non‐affected leg during extension (quadriceps), preoperatively, 6 and 12 months postoperatively, and differences 0–6 and 0–12 months, in all patients that underwent TKA and for patients stratified by anxiety, depression and PC.

Outcome variables	Total population (*n* = 289)	Anxiety (*n* = 60)	Non‐anxiety (*n* = 229)	Depression (*n* = 49)	Non‐depression (*n* = 240)	PC (*n* = 41)	Non‐PC (248)
Preoperative							
SE A leg—extension (Nm/kg)	51.5 ± 24.6	43.3 ± 23.6	53.6 ± 24.5*	43.1 ± 24.3	53.2 ± 24.4*	43.6 ± 26.3	52.8 ± 24.2*
Deficit in SE of A leg—extension (%)	21.8 ± 27.3	28.4 ± 27.6	20.2 ± 27.0*	23.8 ± 32.6	21.4 ± 26.1^†^	28.7 ± 29.1	20.7 ± 26.9^†^
6 months postoperative							
SE A leg—extension (Nm/kg)	53.2 ± 20.6	50.9 ± 22.3	53.7 ± 20.2^†^	48.0 ± 19.6	54.1 ± 20.7^†^	49.9 ± 24.9	53.6 ± 20.0^†^
Deficit in SE of A leg—extension (%)	20.4 ± 26.2	23.2 ± 27.6	19.8 ± 25.9^†^	22.8 ± 30.5	20.0 ± 25.4^†^	23.9 ± 28.1	20.0 ± 26.0^†^
12 months postoperative							
SE A leg—extension (Nm/kg)	61.8 ± 21.4	58.1 ± 21.0	62.7 ± 21.5^†^	55.7 ± 21.9	62.9 ± 21.2^†^	57.3 ± 25.3	62.4 ± 21.0^†^
Deficit in SE of A leg—extension (%)	8.6 ± 53.0	10.9 ± 30.7	8.1 ± 56.8^†^	4.5 ± 62.9	9.4 ± 51.2^†^	14.4 ± 38.4	8.0 ± 54.4^†^
*Δ*SE A leg—extension 0–6 months (Nm/kg)	1.9 ± 17.2	5.6 ± 16.1	1.1 ± 17.3^†^	5.2 ± 19.8	1.4 ± 16.6^†^	2.9 ± 19.2	1.8 ± 16.9^†^
*Δ*SE A leg—flexion 0–6 months (Nm/kg)	9.2 ± 16.9	9.9 ± 12.8	9.0 ± 17.8^†^	11.9 ± 16.2	8.7 ± 17.1^†^	9.9 ± 14.9	9.1 ± 17.2^†^

*Note*: Strength in Nm/kg corrected for bodyweight, deficit in %. All reported in mean (mn) ± standard deviation (SD), independent *t* test used. Forty‐two patients missed outcomes at 6 months postoperatively, and all patients had outcomes preoperatively and at 12 months postoperatively. Significant differences between patients with and without psychological symptoms are indicated by * for *p* < 0.05. Insignificant differences are indicated by † for *p* > 0.05.

Abbreviations: A leg, affected leg; kg, kilograms; Nm, Newton‐metre; PC, pain catastrophizing; SE, strength endurance; TKA, total knee arthroplasty; *Δ*, difference.

### Preoperative differences for depression

Depression patients showed lower extension SE (Table 3 and Appendix S3, 43.1 ± 24.3 vs. 53.2 ± 24.4 Nm/kg, *p* = 0.010, *d* = 0.42 [0.1, 0.7]) and higher TUG times (Table 5 and Appendix S3, 11.2 ± 4.3 vs. 9.7 ± 3.4 s, *p* < 0.021, *d* = −0.44 [−0.7, −0.1]), meaning these patients were slower, compared to non‐depression patients. Strength outcomes and ROM values were similar between depression and non‐depression patients (Tables 2 and 4, Appendix S3).

### Preoperative differences for PC

PC patients had significant lower extension SE (Table 3 and Appendix S3, 43.6 ± 26.3 vs. 52.8 ± 24.2 Nm/kg, *p* = 0.028, *d* = 0.38 [0.0, 0.7]), higher TUG times (Table 5 and Appendix S3, 11.5 ± 4.4 vs. 9.7 ± 3.4 s, *p* = 0.002, *d* = −0.52 [−0.9, −0.2]), and similar strength and ROM values compared to non‐PC patients (Tables 2 and 4, Appendix S3).

### Differences at 6 and 12 months postoperatively

**Figure 3:**
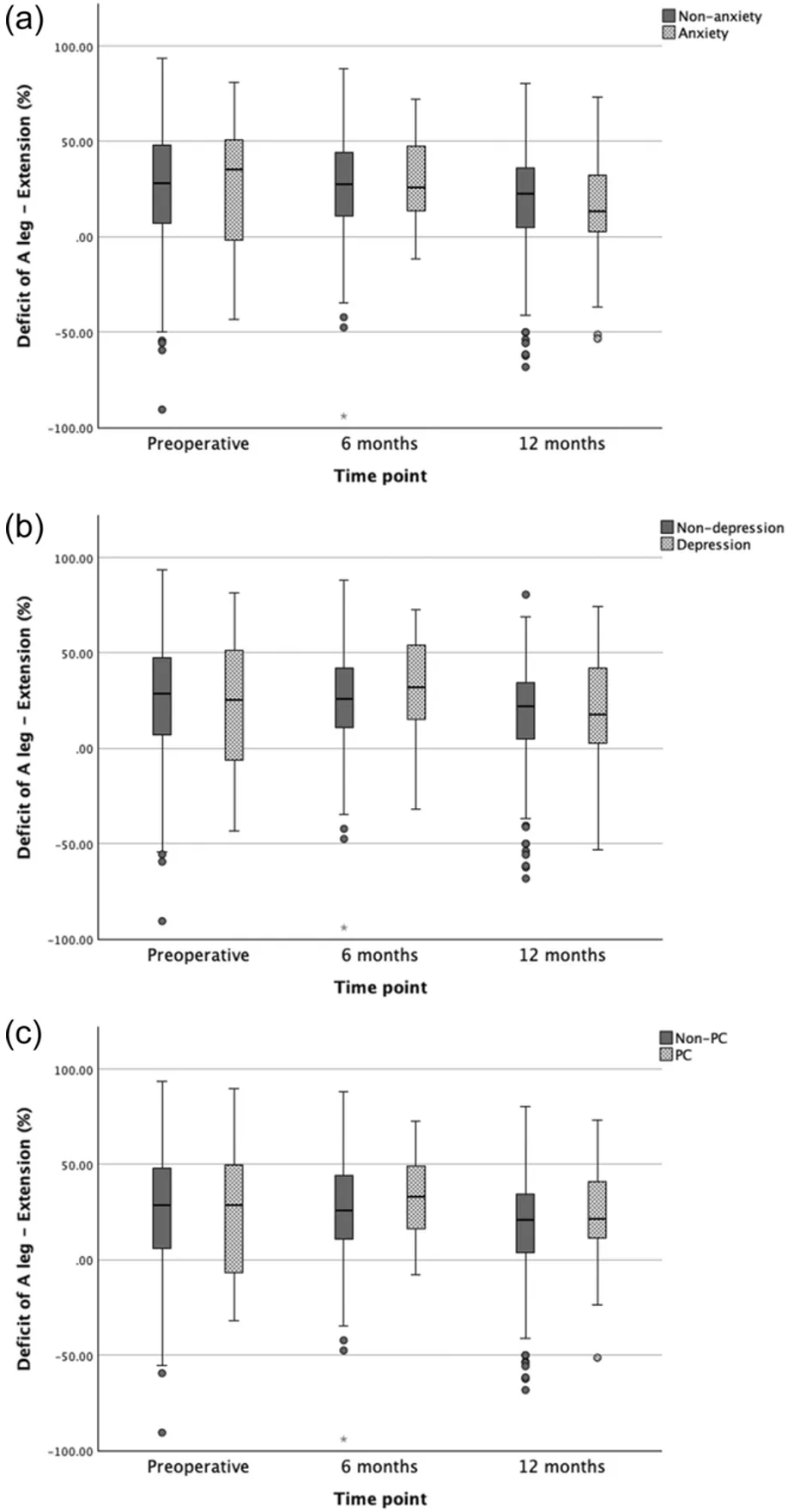
Course of extension strength (maximum strength). Deficits of the affected leg (receiving a TKA) compared to the non‐affected (contralateral) leg, from preoperative to 6‐ and 12‐month follow‐up for (a) anxiety (light‐grey pattern) and non‐anxiety (dark‐grey) patients, (b) depression (light‐grey pattern) and non‐depression (dark‐grey) patients and (c) PC (light‐grey pattern) and non‐PC (dark‐grey) patients. PC, pain catastrophizing; TKA, total knee arthroplasty.

**Figure 4:**
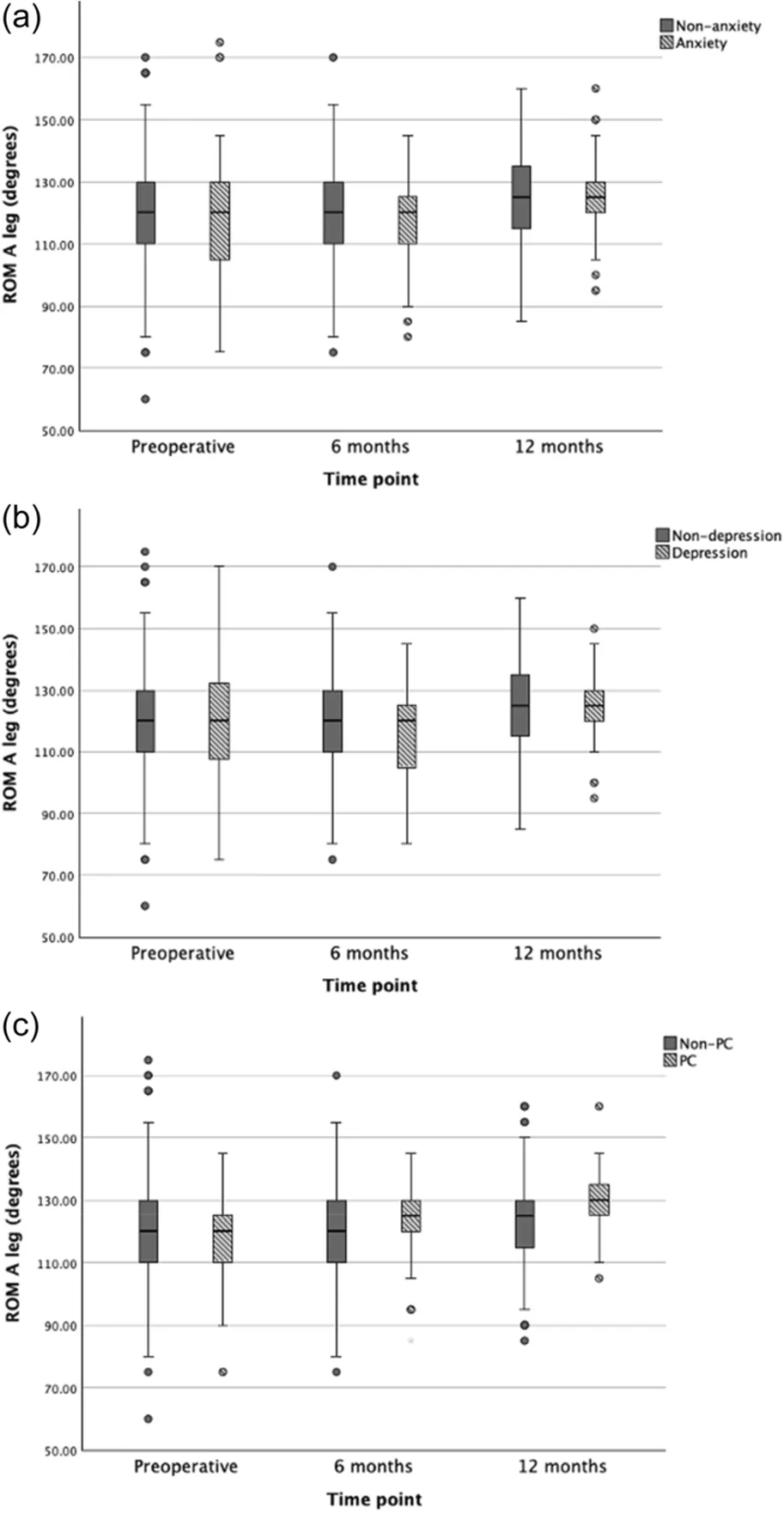
Course of the full range of motion (ROM) of the affected leg (receiving a TKA) from preoperative to 6‐ and 12‐month follow‐up for (a) anxiety (light‐grey pattern) and non‐anxiety (dark‐grey) patients, (b) depression (light‐grey pattern) and non‐depression (dark‐grey) patients and (c) PC (light‐grey pattern) and non‐PC (dark‐grey) patients. PC, pain catastrophizing; TKA, total knee arthroplasty.

At 6 and 12 months postoperatively, all significant differences had resolved, and no differences existed between patients with and without anxiety, depression and/or PC symptoms (Tables 2, 3, 4, 5, Appendix S3, Figures 3 and 4). Multivariable analyses showed similar results (all *p* ≥ 0.157). At 12 months postoperatively, anxiety patients had similar extension strength (Table 2 and Appendix S3, 80.2 ± 33.8 vs. 87.3 ± 30.7 Nm/kg, *p* = 0.225, *d* = 0.30 [−0.1, 0.6]), extension SE (Table 3 and Appendix S3, 58.1 ± 21.0 vs. 62.7 ± 21.5 Nm/kg, *p* = 0.208, *d* = 0.22 [−0.1, 0.6]), ROM (Figure 4a, Table 4 and Appendix S3, 0.2 ± 4.4 vs. 0.5 ± 4.6°, *p* = 0.649, *d* = 0.07 [−0.2, 0.4]) and TUG outcomes (Table 5 and Appendix S3, 8.6 ± 2.9 vs. 8.8 ± 3.3 s, *p* = 0.720, *d* = 0.06 [−0.3, 0.4]) as non‐anxiety patients. These results were similar when adjusted for confounders in multivariable analysis (Appendix S4, for extension strength: *β* = −2.52 [−10.1, 5.0], *p* = 0.511; for extension SE: *β *= 0.19 [−4.7, 5.0], *p* = 0.940; for ROM: *β* = −0.43 [−1.8, 1.0], *p* = 0.537; for TUG: *β* = −0.62 [−1.0, 0.6], *p* = 0.623).

**Table 4:** Objective measurements: ROM of the affected leg during flexion (hamstrings) and extension (quadriceps) preoperatively, 6 and 12 months postoperatively, and differences 0–6 and 0–12 months, in all patients that underwent TKA and for patients stratified by anxiety, depression and PC.

Outcome variables	Total population (*n* = 289)	Anxiety (*n* = 60)	Non‐anxiety (*n* = 229)	Depression (*n* = 49)	Non‐depression (*n* = 240)	PC (*n* = 41)	Non‐PC (248)
Preoperative							
ROM A leg—extension (°)	−0.8 ± 7.1	0.0 ± 7.4	−1.0 ± 7.0^†^	−0.5 ± 7.4	−0.8 ± 7.1^†^	−0.7 ± 7.6	−0.8 ± 7.0^†^
ROM A leg—flexion (°)	120.4 ± 14.9	119.6 ± 17.6	120.6 ± 14.2^†^	120.9 ± 16.6	120.3 ± 14.6^†^	115.6 ± 17.8	121.2 ± 14.3^†^
6 months postoperative							
ROM A leg—extension (°)	−0.5 ± 6.0	−1.1 ± 7.8	−0.4 ± 5.6^†^	−2.3 ± 8.7	−0.2 ± 5.4^†^	−0.9 ± 6.5	−0.5 ± 6.0^†^
ROM A leg—flexion (°)	120.3 ± 12.8	117.0 ± 12.0	121.0 ± 12.9^†^	118.2 ± 12.0	120.6 ± 13.0^†^	122.1 ± 9.3	120.0 ± 13.2^†^
12 months postoperative							
ROM A leg—extension (°)	0.5 ± 4.6	0.19 ± 4.4	0.5 ± 4.6^†^	−0.3 ± 5.5	0.6 ± 4.4^†^	0.6 ± 4.0	0.4 ± 4.6^†^
ROM A leg—flexion (°)	124.1 ± 12.2	124.6 ± 11.4	124.0 ± 12.4^†^	124.7 ± 10.0	124.0 ± 12.6^†^	124.9 ± 12.2	124.0 ± 12.2^†^
*Δ*ROM A leg—extension 0–6 months	0.7 ± 8.5	0.0 ± 8.8	0.8 ± 8.5^†^	−1.1 ± 8.5	1.0 ± 8.5^†^	−0.7 ± 8.9	0.9 ± 8.5^†^
*Δ*ROM A leg—flexion 0–6 months	−0.6 ± 16.1	−2.0 ± 18.3	−0.3 ± 15.7^†^	−3.1 ± 18.2	−0.2 ± 15.8^†^	1.6 ± 14.7	−0.9 ± 16.3^†^
*Δ*ROM A leg—extension 0–12 months	1.3 ± 7.8	0.3 ± 7.9	1.5 ± 7.7^†^	0.1 ± 8.0	1.5 ± 7.7^†^	1.5 ± 7.4	1.2 ± 7.8^†^
*Δ*ROM A leg—flexion 0–12 months	3.5 ± 15.9	3.9 ± 16.3	3.4 ± 15.8^†^	2.4 ± 17.4	3.7 ± 15.6^†^	7.9 ± 19.3	2.8 ± 15.3^†^

*Note*: ROM in degrees (°). All reported in mean (mn) ± standard deviation (SD), independent *t* test used. Forty‐two patients missed outcomes at 6 months postoperatively, and all patients had outcomes preoperatively and at 12 months postoperatively. Insignificant differences are indicated by † for *p* > 0.05.

Abbreviations: A leg, affected leg; PC, pain catastrophizing; ROM, range of motion; TKA, total knee arthroplasty; *Δ*, difference.

**Table 5:** Objective measurements: TUG (s) preoperatively, 6 and 12 months postoperatively, differences 0–6 months and 0–12 months and MCID 0–6 and 0–12 months, in all patients that underwent TKA and for patients stratified by anxiety, depression and PC.

Outcome variables	Total population (*n* = 289)	Anxiety (*n* = 60)	Non‐anxiety (*n* = 229)	Depression (*n* = 49)	Non‐depression (*n* = 240)	PC (*n* = 41)	Non‐PC (248)
Preoperative							
TUG (s)^a^	9.9 ± 3.6	10.5 ± 3.5	9.8 ± 3.6^†^	11.2 ± 4.3	9.7 ± 3.4*	11.5 ± 4.4	9.7 ± 3.4*
6 months postoperative							
TUG (s)^a^	8.9 ± 2.9	8.6 ± 2.1	9.0 ± 3.1^†^	9.2 ± 3.7	8.9 ± 2.8^†^	9.0 ± 2.4	8.9 ± 3.0^†^
12 months postoperative							
TUG (s)^a^	8.8 ± 3.2	8.6 ± 2.9	8.8 ± 3.3^†^	9.6 ± 5.1	8.6 ± 2.7^†^	8.7 ± 3.0	8.8 ± 3.3^†^
*Δ*TUG 0–6 months^a^	−0.9 ± 2.8	−1.2 ± 2.5	−0.8 ± 2.9^†^	−1.6 ± 3.4	−0.7 ± 2.7^†^	−1.2 ± 3.3	−0.8 ± 2.8^†^
*Δ*TUG 0–12 months^a^	−1.1 ± 3.0	−1.5 ± 2.6	−1.0 ± 3.1^†^	−1.3 ± 4.0	−1.1 ± 2.8^†^	−2.4 ± 3.6	−0.9 ± 2.9*
*Δ*MCID 0–6 months^b^	28 (12.3)	6 (15.0)	22 (11.8)^†^	6 (18.2)	22 (11.3)^†^	4 (14.3)	24 (12.1)^†^
*Δ*MCID 0–12 months^b^	42 (15.6)	11 (21.2)	31 (14.3)^†^	11 (25.0)	31 (13.8)^†^	9 (26.5)	33 (14.0)^†^

*Note*: Forty‐two patients missed outcomes at 6 months postoperatively, and all patients had outcomes preoperatively and at 12 months postoperatively. TUG in seconds (s). Significant differences between patients with and without psychological symptoms are indicated by * for *p* < 0.05. Insignificant differences are indicated by † for *p* > 0.05.

Abbreviations: MCID, Minimal Clinical Important Difference; PC, pain catastrophizing; TKA, total knee arthroplasty; TUG, timed up and go; *Δ*, difference.

^a^ Reported in mean (mn) ± standard deviation (SD), independent *t* test used.

^b^ Reported in number (%), *χ* ^2^ test used.

Depression patients also had similar extension strength (Table 2 and Appendix S3, 78.4 ± 37.2 vs. 86.9 ± 30.5 Nm/kg, *p *= 0.180, *d* = 0.284 [−0.1, 0.7]), extension SE (Table 3 and Appendix S3, 55.7 *± *21.9 vs. 62.9 *± *21.2 Nm/kg, *p* = 0.070, *d* = 0.34 [−0.03, 0.7]), ROM (Figure 4b, Table 4 and Appendix S3, −0.3 *± *5.5 vs. 0.6 *± *4.4°, *p* = 0.241, *d* = 0.19 [−0.1, 0.5]) and TUG (Table 5 and Appendix S3, 9.6 *± *5.1 vs. 8.6 *± *2.7 s, *p* = 0.066, *d* = −0.30 [−0.6, 0.02]), compared with non‐depression patients. These results were similar when adjusted for confounders in multivariable analysis (Appendix S4, for extension strength: *β* = −4.32 [−12.5, 3.9], *p* = 0.301; for extension SE: *β* = −1.59 [−6.8, 3.7], *p* = 0.552; for ROM: *β* = −0.96 [−2.4, 0.5], *p* = 0.200; for TUG: *β* = 0.49 [−0.3, 1.3], *p* = 0.246).

Lastly, also PC patients showed similar extension strength (Table 2 and Appendix S3, 78.0 *± *32.9 vs. 87.3 *± *31.0 Nm/kg, *p* = 0.126, *d* = 0.285 [−0.08, 0.7]), extension SE (Table 3 and Appendix S3, 57.3 *± *25.3 vs. 62.3 *± *21.0 Nm/kg, *p* = 0.274, *d* = 0.24 [−0.2, 0.7]), ROM (Figure 4c, Table 4 and Appendix S3, 0.6 ± 4.0 vs. 0.4 ± 4.6°, *p* = 0.846, *d* = −0.04 [−0.4, 0.3]) and TUG (Table 5 and Appendix S3, 8.7 ± 3.0 vs. 8.8 ± 3.3 s, *p* = 0.863, *d* = 0.03 [−0.3, 0.4]), compared with non‐PC patients. These results were similar when adjusted for confounders in multivariable analysis (Appendix S4, for extension strength: *β* = −7.06 [−16.8, 2.7], *p* = 0.157; for extension SE: *β* = −1.89 [−8.2, 4.4], *p* = 0.556; for ROM: *β* = 0.96 [−1.0, 2.4], *p* = 0.415; for TUG: *β* = 0.36 [1.3, 0.6], *p* = 0.458).

Improvements differed only in TUG performances for PC versus non‐PC patients. PC patients had significantly more improvement in TUG times from 0 to 12 months (Table 5 and Appendix S3, PC −2.4 ± 3.6 vs. non‐PC −0.9 ± 2.9 s, *p* = 0.005, *d* = 0.52 [0.2, 0.9]). However, proportions achieving MCID for TUG at 12 months were similar (Table 5 and Appendix S3, 26.5% vs. 14.0% of patients, *p* = 0.062). Lastly, Strength and SE deficits, and proportions attaining strength deficits ≤20% were similar for all groups as well (all *p* ≥ 0.050) (Figure 3a–c, Tables 2 and 3). At 12 months postoperatively, strength deficits ≤20% were attained in similar proportions by anxiety patients and non‐anxiety patients (Figure 3, 28 [60.9%] vs. 93 [44.9%], *p* = 0.050). Similarly, for depression versus non‐depression patients (Figure 3, 21 [55.3%] vs. 100 [46.5%], *p* = 0.319), and for PC versus non‐PC patients (Figure 3, 12 [48.0%] vs. 109 [47.8%], *p* = 0.985).

## DISCUSSION

The main findings of this study were that patients with preoperative symptoms of anxiety, depression and PC had inferior strength and TUG outcomes preoperatively, but that these differences resolved by 6 and 12 months postoperatively. After adjustment for confounders, preoperative psychological symptoms were not independently associated with objectively measured functional outcomes at either 6 or 12 months after TKA. In other words, preoperative symptoms of anxiety, depression and PC are not independently associated with functional outcomes at 6 and 12 months after TKA.

The present study deliberately focused on objectively measured functional outcomes rather than PROMs. It was designed to build upon prior prospective PROM‐based studies, which have consistently demonstrated significant associations between preoperative psychological symptoms and poorer pain, function and quality of life outcomes after TKA [1, 23]. Patient satisfaction was intentionally not included as an outcome measure, as it is a subjective and context‐dependent construct, strongly influenced by expectations, communication and psychosocial factors rather than actual treatment results [29]. The aim of the current study was to complement, rather than replicate, earlier PROM‐focused work and to provide a more comprehensive understanding of how preoperative psychological symptoms relate to objectively measured recovery after TKA.

These findings support the hypothesis of the current study. Although this may appear to contrast with existing literature on the impact of preoperative psychological symptoms on subjective outcomes after TKA (e.g., Knee Injury and Osteoarthritis Outcome Score [KOOS], Oxford Knee Score [OKS], Western Ontario and McMaster Universities Osteoarthritis Index [WOMAC], etc.) [1, 23], it aligns with the theory that patients with anxiety, depression and/or PC symptoms report worse postoperative function, pain and quality of life than is objectively observed. Evidentially, this phenomenon is likely attributable to core mechanisms underlying anxiety, depression and PC, such as ‘central sensitization’ and ‘heightened pain perception’ and highlights the importance of addressing psychological health in preoperative assessments and surgical preparation for optimizing recovery trajectories [6, 9, 36].

The choice for extension strength, ROM and TUG as objective functional outcomes was predominantly based on prior functional performance‐based research within human movement sciences, rehabilitation medicine, geriatrics, neurology and to a lesser extent orthopaedics [20, 30].

Flexion strength and SE were not reported in this study, as the subordinance and irrelevance of flexion strength to extension strength in the rehabilitation of TKA patients is commonly accepted [7, 33]. Extension strength deteriorates more frequently than flexion strength in TKA patients due to less activation, inherent to the nature of knee osteoarthritis and the course of TKA recovery [3, 7, 33]. That is, activities such as walking, running and cycling (extensor strength) are predominantly given up by these patients, whereas flexor strength is merely associated with balance and appears less important in TKA‐recovery [7]. Accordingly, many studies that focus on functional performance in TKA patients left out flexion strength assessments [16, 28].

The TUG is a validated and widely used performance‐based assessment, providing a direct and clinically meaningful evaluation of functional mobility [18]. It integrates multiple components of physical performance, such as balance, strength and coordination, and is sensitive to postoperative improvement over time. Incorporating such standardized, objective metrics helps bridge the gap between perceived and measured recovery and represents an important step toward a more complete, evidence‐based evaluation of TKA outcomes.

To our knowledge, only three previous studies have examined the relationship between psychological health and objectively measured functional outcomes after TKA [14, 16, 22]. Hayashi et al. focused exclusively on the association between PC symptoms and TUG and gait speeds, at 14 days postoperatively, reporting a significant association between more PC symptoms and reductions in physical performance (TUG) after TKA [16]. This aligns with the current study showing greater TUG improvements (clinically insignificant) over 12‐month follow‐up in PC patients (Table 5). These results are understandable in view of the higher preoperative TUG times for patients with depression and PC symptoms, implying more room for improvement in these patients. Hanusch et al. found no association between anxiety or depression (HADS) and ROM‐outcomes 12 months after TKA [14], consistent with the current study. Lastly, Lavernia et al. reported no association between psychological distress (SF‐36 mental‐component score) and ROM‐outcomes up to 2 years after TKA, conform trends in the current study [22]. Notably, this study is the first to assess the association between psychological distress and extension strength outcomes in TKA patients. Therefore, this study is the first to show that no differences exist between patients with and without psychological distress for the important TKA parameter ‘extension strength’.

PC patients showed greater TUG improvements than non‐PC patients over 12 months, aligning with subjective outcome studies reporting greater function PROM improvements in these groups after TKA [1, 23]. However, despite greater improvements in patients with psychological symptoms, they start and end at significantly lower (or similar) levels of objective and subjective function, pain and higher dissatisfaction rates than patients without these psychological symptoms. Thus, more improvement in function for anxiety/depression/PC patients might be due to more ‘room for improvement’ on the scale.

There were several limitations. Psychological assessment was based solely on preoperative PROMs, without postoperative reassessment. This is a significant limiting factor of this studies' methodology for investigating the association between psychological symptoms and objective functional outcomes, because potential changes in psychological status after surgery were not captured, and their possible influence on functional recovery could not be evaluated in this study. Moreover, no clinical evaluations by a psychologist or psychiatrist were done, potentially underestimating psychological distress [31]. Furthermore, follow‐up was limited to 12 months; however, most functional improvement occurs within the first year after TKA [25]. Additionally, while 289 patients were included, proportions with anxiety, depression and PC symptoms were small, potentially reducing statistical power for some comparisons. Also, the relatively high proportion of patients who did not attend the functional performance assessments may have introduced selection bias, particularly related to patient motivation. Although we minimized logistical barriers by scheduling all functional performance assessments to coincide with routine pre‐ and postoperative clinical appointments, participation still required an additional degree of willingness and engagement. Therefore, some self‐selection based on motivation cannot be excluded, which may limit the generalizability of the findings to the broader TKA population. Furthermore, a non‐inferiority design or a much larger sample would be needed to confirm the absence of group differences with certainty, and that it is not due to a lack of statistical power. Nonetheless, this is the largest study to date assessing the influence of psychological factors on strength, ROM and TUG outcomes after TKA.

Future research should focus on exploring these trends in larger cohorts with extended follow‐up and implementation of psychological support in the perioperative pathway. Addressing psychological health within the perioperative pathway is essential in optimizing patient‐perceived recovery after TKA, as the perception and objective measurement of pain and function do not align in patients with psychological symptoms.

## CONCLUSION

Although preoperative psychological symptoms are associated with poorer objective outcomes, these differences resolve by 6–12 months postoperative follow‐up. After 1 year, similar objective outcomes are attained for patients with symptoms of anxiety, depression or PC. Interpreting this in the bigger scope, the importance of integrating psychological support into the perioperative pathway to align subjective and objective outcomes is underlined.

## AUTHOR CONTRIBUTIONS

Margot B. Aalders and Jelle P. van der List designed the research question, were responsible for the study conception and design, data analysis and interpretation, drafting and revising the manuscript and approving the final version. Joyce L. Benner provided essential biomechanical expertise from her background in human movement sciences, and supported study design and designing the research question. She also contributed to the data acquisition, analysis and interpretation, created figures, and participated in drafting, revising and approving the manuscript. Stef Daniel assisted in presenting preliminary findings at conferences in the absence of the primary authors, contributed to critical manuscript revision and approved the final version. Lucien C. M. Keijser, Gino M. M. J. Kerkhoffs, Job N. Doornberg and Ruurd L. Jaarsma contributed to the study design, provided clinical expertise and supervision, critically revised the manuscript and approved the final version.

## CONFLICT OF INTEREST STATEMENT

The authors declare no conflicts of interest.

## ETHICS STATEMENT

Ethical approval for this study was obtained from the Northwest Hospital Group (approval number: L019‐075). This study concerned non Medical Research Involving Human Subjects Act (WMO) research and therefore did not require formal Medical Ethics Committee approval under Dutch law. In accordance with national and institutional regulations, institutional review board (IRB) approval was obtained prior to study initiation from the Institutional Review Board of Noordwest Ziekenhuisgroep, Alkmaar, the Netherlands (IRB reference: L019‐075/FOLLOW). All procedures were conducted in accordance with the principles of the Declaration of Helsinki. Written informed consent was obtained from all individual participants included in the study.

## Supporting information

Supporting information.

Supporting information.

Supporting information.

Supporting information.

## Data Availability

Data are available on request from the authors.
