# Supplementary material for: Objective functional performance 1 year after total knee arthroplasty does not differ for patients with symptoms of anxiety, depression or pain catastrophizing: A prospective study of 289 patients
Source: J Exp Orthop. 2026 Jan 19;13(1):e70645. doi: 10.1002/jeo2.70645 (PMC12814217; doi:10.1002/jeo2.70645)
Supplement: Supplementary file 2 — Supporting information. [file JEO2-13-e70645-s003.pdf]

Pain Catastrophizing Scale

Full name of the patient: _______________ Date accomplished: _______________

Age: ________ Gender: ☐ Male ☐ Female ☐ Non-Binary

Full name of assessor: _______________

Instructions: Hello! We would like to help find ways to assuage the pain you are feeling, but first, we would like to gauge how the pain you are feeling is affecting you. Below are 13 items and all you need to do is to tick the answer that best represents how you feel based on the item.

**RATING**	**0**	**1**	**2**	**3**	**4**
**MEANING**	Not at all	To a slight degree	To a moderate degree	To a great degree	All the time

When I'm in pain...

Number	Statement	Rating
1	I worry all the time about whether the pain will end.	
2	I feel I can't go on.	
3	It's terrible and I think it's never going to get any better	
4	It's awful and I feel that it overwhelms me	
5	I feel I can't stand it anymore	
6	I become afraid that the pain will get worse	
7	I keep thinking of other painful events	
8	I anxiously want the pain to go away	
9	I can't seem to keep it out of my mind	
10	I keep thinking about how much it hurts	
11	I keep thinking about how badly I want the pain to stop	
12	There's nothing I can do to reduce the intensity of the pain	
13	I wonder whether something serious may happen.	

Total Score: _______________

Copyright 1995 Michael J.L. Sullivan. Source: Sullivan MJL, Bishop S, Pivik J. The pain catastrophizing scale: development and validation. Psychol Assess, 1995, 7: 524-532
