# Supplementary material for: Objective functional performance 1 year after total knee arthroplasty does not differ for patients with symptoms of anxiety, depression or pain catastrophizing: A prospective study of 289 patients
Source: J Exp Orthop. 2026 Jan 19;13(1):e70645. doi: 10.1002/jeo2.70645 (PMC12814217; doi:10.1002/jeo2.70645)
Supplement: Supplementary file 3 — Supporting information. [file JEO2-13-e70645-s001.docx]

**APPENDIX 3:** Baseline and primary outcome supplemental tables 1-5, including P-values

| **Table 1.** Baseline characteristics for all patients scheduled for TKA and for patients stratified by anxiety, depression and pain catastrophizing | | | | | | | | | | |
| --- | --- | --- | --- | --- | --- | --- | --- | --- | --- | --- |
| Baseline variables | Total population  (n = 289) | Anxiety  (n = 60) | Non-anxiety  (n = 229) | P-value | Depression  (n = 49) | Non-depression  (n = 240) | P-value | PC  (n = 41) | Non-PC  (248) | P-value |
| Age (years) ^a^ | 71.0 (65-76) | 70.5 (61-75) | 71.0 (65-76) | 0.121 | 70.0 (61-75) | 71.0 (65-76) | 0.144 | 68.0 (58-74) | 72.0 (65-76) | **0.002** |
| Female gender ^b^ | 160 (55.4) | 41 (68.3) | 119 (52.0) | **0.023** | 31 (63.3) | 129 (53.8) | 0.222 | 26 (63.4) | 134 (54.0) | 0.263 |
| BMI (kg/m^2^) ^c^ | 28.6 ± 4.3 | 29.2 ± 4.4 | 28.5 ± 4.3 | 0.287 | 29.2 ± 5.4 | 28.5 ± 4.1 | 0.433 | 30.5 ± 4.6 | 28.3 ± 4.2 | **0.002** |
| Smoking ^b^ | 17 (5.9) | 5 (8.3) | 12 (5.2) | 0.365 | 4 (8.2) | 13 (5.4) | 0.456 | 1 (2.4) | 16 (6.5) | 0.312 |
| ASA I/II ^b^ | 214 (74.0) | 41 (68.3) | 173 (75.5) | 0.257 | 29 (59.2) | 185 (77.1) | **0.009** | 31 (75.6) | 183 (73.8) | 0.806 |
| Mean HADS-anxiety score ^a^ | 4.0 (2-7) | 10.0 (8-12) | 3.0 (2-5) | **0.000** | - | - |  | - | - |  |
| Mean HADS-depression score ^a^ | 3.0 (2-6) | - | - |  | 10.0 (8-11) | 3.0 (1-4) | <**0.001** | - | - |  |
| Mean PCS score ^a^ | 11.0 (4-21) | - | - |  | - | - |  | 35.0 (31-43) | 9.0 (3-16) | **<0.001** |
| Right side ^b^ | 142 (49.1) | 33 (55.0) | 109 (47.6) | 0.307 | 30 (61.2) | 112 (46.7) | 0.063 | 20 (48.8) | 122 (49.2) | 0.961 |
| Physical therapy pre-operatively ^b^ | 90 (31.1) | 20 (50.0) | 70 (43.2) | 0.439 | 15 (48.4) | 75 (43.9) | 0.641 | 14 (46.7) | 76 (44.2) | 0.801 |
| Perioperative variables |  |  |  |  |  |  |  |  |  |  |
| Medial parapatellar approach ^b^ | 265 (91.7) | 57 (95.0) | 208 (90.8) | 0.297 | 45 (91.8) | 220 (91.7) | 0.969 | 41 (100.0) | 224 (90.3) | **0.038** |
| Patellar resurfacing ^b^ | 54 (18.7) | 10 (16.7) | 44 (19.2) | 0.652 | 8 (16.3) | 46 (19.2) | 0.642 | 7 (17.1) | 47 (19.0) | 0.775 |
| Surgery duration (minutes) ^a^ | 72.0 (61-83) | 69.0 (59-90) | 72.0 (62-81) | 0.770 | 69.0 (60-89) | 72.0 (62-81) | 0.811 | 72.0 (60-84) | 72.0 (61-82) | 0.762 |
| Estimated blood loss (mL) ^a^ | 200.0 (100-300) | 200.0 (100-325) | 200.0 (100-300) | 0.737 | 200.0 (100-300) | 200.0 (100-300) | 0.992 | 175.0 (100-300) | 200.0 (100-300) | 0.681 |
| Length of stay (days) ^a^ | 1.0 (1-2) | 1.0 (1-3) | 1.0 (1-2) | 0.210 | 1.0 (1-3) | 1.0 (1-2) | **0.049** | 1.0 (1-2) | 1.0 (1-2) | 0.170 |
| Length of stay ≥3 days ^b^ | 51 (17.6) | 15 (25.4) | 36 (15.9) | 0.087 | 14 (28.6) | 37 (15.6) | **0.031** | 9 (22.5) | 42 (17.1) | 0.406 |
| ^a^, reported in median with interquartile range (lower quartile – upper quartile). ^b^, reported in number (%). ^c^, reported in mean ± standard deviation.  n, number of patients; BMI, body mass index; ASA, American Society of Anaesthesiologists physical status classification. | | | | | | | | | | |

| **Table 2.** Objective measurements: Maximum Strength (Peak Torque corrected for bodyweight and measured at 60 degrees/second) of Affected Leg & the percentage Strength-deficit of Affected leg compared to the Non-Affected leg during Extension (quadriceps), preoperatively, 6 months and 12 months postoperatively, and differences 0-6 months and 0-12 months, in all patients that underwent TKA and for patients stratified by anxiety, depression and pain catastrophizing. | | | | | | | | | | | |
| --- | --- | --- | --- | --- | --- | --- | --- | --- | --- | --- | --- |
| Outcome variables | | Total population  (n = 289) | Anxiety  (n = 60) | Non-anxiety  (n = 229) | *p*-value | Depression  (n = 49) | Non-depression  (n = 240) | *p*-value | PC  (n = 41) | Non-PC  (248) | *p*-value |
| Preoperative | |  |  |  |  |  |  |  |  |  |  |
|  | Strength A leg - Extension (Nm/kg) | 69.0 ± 35.1 | 60.7 ± 35.0 | 71.1 ± 34.9 | **0.048** | 60.5 ± 35.4 | 70.8 ± 34.8 | 0.066 | 60.2 ± 39.3 | 70.5 ± 34.2 | 0.085 |
|  | Deficit of A leg - Extension (%) | 24.2 ± 37.0 | 25.4 ± 34.7 | 24.0 ± 37.6 | 0.797 | 19.1 ± 52.5 | 25.3 ± 33.0 | 0.434 | 26.7 ± 35.0 | 23.8 ± 37.4 | 0.658 |
| 6 months postoperative | |  |  |  |  |  |  |  |  |  |  |
|  | Strength A leg - Extension (Nm/kg) | 71.7 ± 28.7 | 70.5 ± 33.5 | 72.0 ± 27.7 | 0.761 | 64.6 ± 29.2 | 72.9 ± 28.6 | 0.127 | 67.8 ± 34.4 | 72.2 ± 28.0 | 0.475 |
|  | Deficit of A leg - Extension (%) | 23.6 ± 39.8 | 29.6 ± 22.2 | 22.2 ± 42.7 | 0.277 | 31.5 ± 27.2 | 22.2 ± 41.5 | 0.208 | 32.7 ± 21.1 | 22.4 ± 41.5 | 0.214 |
| 12 months postoperative | |  |  |  |  |  |  |  |  |  |  |
|  | Strength A leg - Extension (Nm/kg) | 86.0 ± 31.3 | 80.2 ± 33.8 | 87.3 ± 30.7 | 0.184 | 78.4 ± 32.9 | 87.3 ± 31.0 | 0.126 | 78.0 ± 37.2 | 86.9 ± 30.5 | 0.180 |
|  | Deficit of A leg - Extension (%) | 14.5 ± 46.3 | 14.8 ± 27.6 | 14.4 ± 49.6 | 0.957 | 12.7 ± 47.0 | 14.8 ± 46.3 | 0.799 | 21.9 ± 26.5 | 13.6 ± 48.0 | 0.400 |
| Δ Strength A leg Extension 0-6 months (Nm/kg) | | 2.7 ± 28.7 | 5.4 ± 25.0 | 2.1 ± 29.4 | 0.509 | 4.3 ± 31.9 | 2.4 ± 28.1 | 0.737 | 2.7 ± 28.3 | 2.7 ± 28.8 | 0.996 |
| Δ Strength A leg Extension 0-12 months (Nm/kg) | | 15.7 ± 29.3 | 16.1 ± 27.7 | 15.7 ± 29.7 | 0.932 | 17.7 ± 31.4 | 15.4 ± 29.0 | 0.673 | 11.5 ± 29.3 | 16.2 ± 29.4 | 0.456 |
| A leg, Affected leg; TKA, total knee arthroplasty; PC, pain catastrophizing; Nm, Newton-meter; kg, kilograms; Δ, difference.  Strength in Nm/kg corrected for bodyweight, Deficit in %. All reported in mean (mn) ± standard deviation (SD).  42 patients missed outcomes at 6 months postoperatively, and all patients had outcomes preoperatively and at 12 months postoperatively. | | | | | | | | | | | |

| **Table 3.** Objective measurements: Strength Endurance (Peak Torque corrected for bodyweight and measured at 180 degrees/second) of Affected Leg & Strength-deficit of Affected leg compared to the Non-Affected leg during Extension (quadriceps), preoperatively, 6 months and 12 months postoperatively, and differences 0-6 months and 0-12 months, in all patients that underwent TKA and for patients stratified by anxiety, depression and pain catastrophizing. | | | | | | | | | | | |
| --- | --- | --- | --- | --- | --- | --- | --- | --- | --- | --- | --- |
| Outcome variables | | Total population  (n = 289) | Anxiety  (n = 60) | Non-anxiety  (n = 229) | P-value | Depression  (n = 49) | Non-depression  (n = 240) | P-value | PC  (n = 41) | Non-PC  (248) | P-value |
| Preoperative | |  |  |  |  |  |  |  |  |  |  |
|  | SE A leg - Extension (Nm/kg) | 51.5 ± 24.6 | 43.3 ± 23.6 | 53.6 ± 24.5 | **0.005** | 43.1 ± 24.3 | 53.2 ± 24.4 | **0.010** | 43.6 ± 26.3 | 52.8 ± 24.2 | **0.028** |
|  | Deficit in SE of A leg - Extension (%) | 21.8 ± 27.3 | 28.4 ± 27.6 | 20.2 ± 27.0 | **0.041** | 23.8 ± 32.6 | 21.4 ± 26.1 | 0.634 | 28.7 ± 29.1 | 20.7 ± 26.9 | 0.084 |
| 6 months postoperative | |  |  |  |  |  |  |  |  |  |  |
|  | SE A leg - Extension (Nm/kg) | 53.2 ± 20.6 | 50.9 ± 22.3 | 53.7 ± 20.2 | 0.438 | 48.0 ± 19.6 | 54.1 ± 20.7 | 0.117 | 49.9 ± 24.9 | 53.6 ± 20.0 | 0.394 |
|  | Deficit in SE of A leg - Extension (%) | 20.4 ± 26.2 | 23.2 ± 27.6 | 19.8 ± 25.9 | 0.439 | 22.8 ± 30.5 | 20.0 ± 25.4 | 0.567 | 23.9 ± 28.1 | 20.0 ± 26.0 | 0.467 |
| 12 months postoperative | |  |  |  |  |  |  |  |  |  |  |
|  | SE A leg - Extension (Nm/kg) | 61.8 ± 21.4 | 58.1 ± 21.0 | 62.7 ± 21.5 | 0.208 | 55.7 ± 21.9 | 62.9 ± 21.2 | 0.070 | 57.3 ± 25.3 | 62.4 ± 21.0 | 0.274 |
|  | Deficit in SE of A leg - Extension (%) | 8.6 ± 53.0 | 10.9 ± 30.7 | 8.1 ± 56.8 | 0.750 | 4.5 ± 62.9 | 9.4 ± 51.2 | 0.606 | 14.4 ± 38.4 | 8.0 ± 54.4 | 0.566 |
| Δ SE A leg - Extension 0-6 months (Nm/kg) | | 1.9 ± 17.2 | 5.6 ± 16.1 | 1.1 ± 17.3 | 0.136 | 5.2 ± 19.8 | 1.4 ± 16.6 | 0.233 | 2.9 ± 19.2 | 1.8 ± 16.9 | 0.759 |
| Δ SE A leg - Flexion 0-6 months (Nm/kg) | | 9.2 ± 16.9 | 9.9 ± 12.8 | 9.0 ± 17.8 | 0.771 | 11.9 ± 16.2 | 8.7 ± 17.1 | 0.314 | 9.9 ± 14.9 | 9.1 ± 17.2 | 0.826 |
| A leg, Affected leg; TKA, total knee arthroplasty; PC, pain catastrophizing; Nm, Newton-meter; kg, kilograms; Δ, difference.  Strength in Nm/kg corrected for bodyweight, Deficit in %. All reported in mean (mn) ± standard deviation (SD).  42 patients missed outcomes at 6 months postoperatively, and all patients had outcomes preoperatively and at 12 months postoperatively. | | | | | | | | | | | |

| **Table 4.** Objective measurements: Range of Motion (ROM) of the Affected Leg during Flexion (hamstrings) & Extension (quadriceps) preoperatively, 6 months and 12 months postoperatively, and differences 0-6 months and 0-12 months, in all patients that underwent TKA and for patients stratified by anxiety, depression and pain catastrophizing. | | | | | | | | | | | |
| --- | --- | --- | --- | --- | --- | --- | --- | --- | --- | --- | --- |
| Outcome variables | | Total population  (n = 289) | Anxiety  (n = 60) | Non-anxiety  (n = 229) | P-value | Depression  (n = 49) | Non-depression  (n = 240) | P-value | PC  (n = 41) | Non-PC  (248) | P-value |
| Preoperative | |  |  |  |  |  |  |  |  |  |  |
|  | ROM A leg - Extension (°) | -0.8 ± 7.1 | 0.0 ± 7.4 | -1.0 ± 7.0 | 0.353 | -0.5 ± 7.4 | -0.8 ± 7.1 | 0.774 | -0.7 ± 7.6 | -0.8 ± 7.0 | 0.966 |
|  | ROM A leg - Flexion (°) | 120.4 ± 14.9 | 119.6 ± 17.6 | 120.6 ± 14.2 | 0.641 | 120.9 ± 16.6 | 120.3 ± 14.6 | 0.784 | 115.6 ± 17.8 | 121.2 ± 14.3 | 0.062 |
| 6 months postoperative | |  |  |  |  |  |  |  |  |  |  |
|  | ROM A leg - Extension (°) | -0.5 ± 6.0 | -1.1 ± 7.8 | -0.4 ± 5.6 | 0.533 | -2.3 ± 8.7 | -0.2 ± 5.4 | 0.169 | -0.9 ± 6.5 | -0.5 ± 6.0 | 0.733 |
|  | ROM A leg - Flexion (°) | 120.3 ± 12.8 | 117.0 ± 12.0 | 121.0 ± 12.9 | 0.063 | 118.2 ± 12.0 | 120.6 ± 13.0 | 0.320 | 122.1 ± 9.3 | 120.0 ± 13.2 | 0.408 |
| 12 months postoperative | |  |  |  |  |  |  |  |  |  |  |
|  | ROM A leg - Extension (°) | 0.5 ± 4.6 | 0.19 ± 4.4 | 0.5 ± 4.6 | 0.649 | -0.3 ± 5.5 | 0.6 ± 4.4 | 0.241 | 0.6 ± 4.0 | 0.4 ± 4.6 | 0.846 |
|  | ROM A leg - Flexion (°) | 124.1 ± 12.2 | 124.6 ± 11.4 | 124.0 ± 12.4 | 0.750 | 124.7 ± 10.0 | 124.0 ± 12.6 | 0.759 | 124.9 ± 12.2 | 124.0 ± 12.2 | 0.717 |
| Δ ROM A leg Extension 0-6 months | | 0.7 ± 8.5 | 0.0 ± 8.8 | 0.8 ± 8.5 | 0.576 | -1.1 ± 8.5 | 1.0 ± 8.5 | 0.194 | -0.7 ± 8.9 | 0.9 ± 8.5 | 0.361 |
| Δ ROM A leg Flexion 0-6 months | | -0.6 ± 16.1 | -2.0 ± 18.3 | -0.3 ± 15.7 | 0.525 | -3.1 ± 18.2 | -0.2 ± 15.8 | 0.329 | 1.6 ± 14.7 | -0.9 ± 16.3 | 0.444 |
| Δ ROM A leg Extension 0-12 months | | 1.3 ± 7.8 | 0.3 ± 7.9 | 1.5 ± 7.7 | 0.323 | 0.1 ± 8.0 | 1.5 ± 7.7 | 0.263 | 1.5 ± 7.4 | 1.2 ± 7.8 | 0.857 |
| Δ ROM A leg Flexion 0-12 months | | 3.5 ± 15.9 | 3.9 ± 16.3 | 3.4 ± 15.8 | 0.851 | 2.4 ± 17.4 | 3.7 ± 15.6 | 0.620 | 7.9 ± 19.3 | 2.8 ± 15.3 | 0.079 |
| ROM, Range Of Motion; A leg, Affected leg; TKA, total knee arthroplasty; PC, pain catastrophizing; Δ, difference.  ROM in degrees (°), Deficit in %. All reported in mean (mn) ± standard deviation (SD).  42 patients missed outcomes at 6 months postoperatively, and all patients had outcomes preoperatively and at 12 months postoperatively. | | | | | | | | | | | |

| **Table 5.** Objective measurements: Timed Up and Go (seconds) preoperatively, 6 months and 12 months postoperatively, differences 0-6 months and 0-12 months and MCID 0-6 months and 0-12 months, in all patients that underwent TKA and for patients stratified by anxiety, depression and pain catastrophizing. | | | | | | | | | | | |
| --- | --- | --- | --- | --- | --- | --- | --- | --- | --- | --- | --- |
| Outcome variables | | Total population  (n = 289) | Anxiety  (n = 60) | Non-anxiety  (n = 229) | P-value | Depression  (n = 49) | Non-depression  (n = 240) | P-value | PC  (n = 41) | Non-PC  (248) | P-value |
| Preoperative | |  |  |  |  |  |  |  |  |  |  |
|  | TUG (s)^a^ | 9.9 ± 3.6 | 10.5 ± 3.5 | 9.8 ± 3.6 | 0.174 | 11.2 ± 4.3 | 9.7 ± 3.4 | **0.021** | 11.5 ± 4.4 | 9.7 ± 3.4 | **0.002** |
| 6 months postoperative | |  |  |  |  |  |  |  |  |  |  |
|  | TUG (s) ^a^ | 8.9 ± 2.9 | 8.6 ± 2.1 | 9.0 ± 3.1 | 0.450 | 9.2 ± 3.7 | 8.9 ± 2.8 | 0.630 | 9.0 ± 2.4 | 8.9 ± 3.0 | 0.951 |
| 12 months postoperative | |  |  |  |  |  |  |  |  |  |  |
|  | TUG (s) ^a^ | 8.8 ± 3.2 | 8.6 ± 2.9 | 8.8 ± 3.3 | 0.720 | 9.6 ± 5.1 | 8.6 ± 2.7 | 0.223 | 8.7 ± 3.0 | 8.8 ± 3.3 | 0.863 |
| Δ TUG 0-6 months^a^ | | -0.9 ± 2.8 | -1.2 ± 2.5 | -0.8 ± 2.9 | 0.388 | -1.6 ± 3.4 | -0.7 ± 2.7 | 0.102 | -1.2 ± 3.3 | -0.8 ± 2.8 | 0.516 |
| Δ TUG 0-12 months ^a^ | | -1.1 ± 3.0 | -1.5 ± 2.6 | -1.0 ± 3.1 | 0.242 | -1.3 ± 4.0 | -1.1 ± 2.8 | 0.622 | -2.4 ± 3.6 | -0.9 ± 2.9 | **0.005** |
| Δ MCID 0-6 months ^b^ | | 28 (12.3) | 6 (15.0) | 22 (11.8) | 0.572 | 6 (18.2) | 22 (11.3) | 0.269 | 4 (14.3) | 24 (12.1) | 0.737 |
| Δ MCID 0-12 months ^b^ | | 42 (15.6) | 11 (21.2) | 31 (14.3) | 0.220 | 11 (25.0) | 31 (13.8) | 0.061 | 9 (26.5) | 33 (14.0) | 0.062 |
| TUG, Timed Up and Go; MCID, Minimal Clinical Important Difference; TKA, total knee arthroplasty; PC, pain catastrophizing; Δ, difference. TUG in s (seconds).  ^a^ Reported in mean (mn) ± standard deviation (SD). ^b^ Reported in Number (%).  42 patients missed outcomes at 6 months postoperatively, and all patients had outcomes preoperatively and at 12 months postoperatively. | | | | | | | | | | | |
