# Supplementary material for: Objective functional performance 1 year after total knee arthroplasty does not differ for patients with symptoms of anxiety, depression or pain catastrophizing: A prospective study of 289 patients
Source: J Exp Orthop. 2026 Jan 19;13(1):e70645. doi: 10.1002/jeo2.70645 (PMC12814217; doi:10.1002/jeo2.70645)
Supplement: Supplementary file 4 — Supporting information. [file JEO2-13-e70645-s004.docx]

**APPENDIX 4:** Univariable and Multivariable Regression Analyses

| **Supplemental Table 1.** Regression models for Extension Strength & *Anxiety* with adjustment for baseline confounders and baseline Extension Strength scores. | | | | | |
| --- | --- | --- | --- | --- | --- |
| **Outcome** | **Predictor** | **sign** | **Beta** | **95%-CI** | ***P-*value** |
| Extension Strength  at 6 months | ^1^ Anxiety yes/no |  | -1.515 | -11.3, 8.93 | 0.761 |
|  | ^2^ Anxiety yes/no, baseline confounders^a^ | Age, sex, BMI | -0.084 | -8.7, 8.5 | 0.985 |
|  | ^2^ Anxiety yes/no, baseline confounders^a^, baseline score | Baseline function | 1.405 | -6.3, 9.1 | 0.719 |
|  | | | | | |
| Extension Strength  at 12 months | ^1^ Anxiety yes/no |  | -7.029 | -17.4, 3.4 | 0.184 |
|  | ^2^ Anxiety yes/no, baseline confounders^a^ | Age, sex, BMI | -3.661 | -12.1, 4.8 | 0.392 |
|  | ^2^ Anxiety yes/no, baseline confounders^a^, baseline score | Age, sex, BMI, baseline function | -2.521 | -10.1, 5.0 | 0.511 |
| ^1^ Univariate regression analysis  ^2^ Multivariate regression analysis  ^a^ Baseline confounders include Age, Gender, ASA I/II, BMI, and Surgical approach. | | | | | |

| **Supplemental Table 2.** Regression models for Extension Strength Deficits of the affected leg compared to the non-affected leg & *Anxiety* with adjustment for baseline confounders and baseline Extension Strength Deficit scores. | | | | | |
| --- | --- | --- | --- | --- | --- |
| **Outcome** | **Predictor** | **sign** | **Beta** | **95%-CI** | ***P-*value** |
| Extension Strength Deficits  at 6 months | ^1^ Anxiety yes/no |  | 7.403 | -6.0, 20.8 | 0.277 |
|  | ^2^ Anxiety yes/no, baseline confounders^a^ | age | 4.520 | -9.1, 18.1 | 0.514 |
|  | ^2^ Anxiety yes/no, baseline confounders^a^, baseline score | Age, Baseline function | 4.306 | -9.3, 17.9 | 0.534 |
|  | | | | | |
| Extension Strength Deficits  at 12 months | ^1^ Anxiety yes/no |  | 0.406 | -14.5, 15.3 | 0.957 |
|  | ^2^ Anxiety yes/no, baseline confounders^a^ | BMI | -0.738 | -15.9, 14.4 | 0.924 |
|  | ^2^ Anxiety yes/no, baseline confounders^a^, baseline score | BMI, baseline function | -1.199 | -16.4, 14.0 | 0.876 |
| ^1^ Univariate regression analysis  ^2^ Multivariate regression analysis  ^a^ Baseline confounders include Age, Gender, ASA I/II, BMI, and Surgical approach. | | | | | |

| **Supplemental Table 3.** Regression models for Extension Strength & *Depression* with adjustment for baseline confounders and baseline Extension Strength score. | | | | | |
| --- | --- | --- | --- | --- | --- |
| **Outcome** | **Predictor** | **Sign** | **Beta** | **95%-CI** | ***P-*value** |
| Extension Strength  at 6 months | ^1^ Depression yes/no |  | -8.287 | -18.9, 2.4 | 0.127 |
|  | ^2^ Depression yes/no, baseline confounders^a^ | Age, sex, BMI | -7.262 | -16.6, 2.0 | 0.125 |
|  | ^2^ Depression yes/no, baseline confounders^a^, baseline score | Sex, BMI, baseline function | -3.648 | -12.0, 4.7 | 0.388 |
|  | | | | | |
| Extension Strength  at 12 months | ^1^ Depression yes/no |  | -8.891 | -20.3, 2.5 | 0.126 |
|  | ^2^ Depression yes/no, baseline confounders^a^ | Age, sex, BMI | -8.594 | -17.8, 0.6 | 0.067 |
|  | ^2^Depression yes/no, baseline confounders^a^, baseline score | Age, sex, BMI, baseline function | -4.321 | -12.5, 3.9 | 0.301 |
| ^1^ Univariate regression analysis  ^2^ Multivariate regression analysis  ^a^ Baseline confounders include Age, Gender, ASA I/II, BMI, and Surgical approach. | | | | | |

| **Supplemental Table 4.** Regression models for Extension Strength Deficits of the affected leg compared to the non-affected leg & *Depression* with adjustment for baseline confounders and baseline Extension Strength Deficit score. | | | | | |
| --- | --- | --- | --- | --- | --- |
| **Outcome** | **Predictor** | **sign** | **Beta** | **95%-CI** | ***P-*value** |
| Extension Strength Deficit  at 6 months | ^1^ Depression yes/no |  | 9.322 | -5.2, 23.8 | 0.208 |
|  | ^2^ Depression yes/no, baseline confounders^a^ | Age | 7.065 | -7.7, 21.8 | 0.347 |
|  | ^2^ Depression yes/no, baseline confounders^a^, baseline score | Age, baseline function | 7.110 | -7.5, 21.8 | 0.340 |
|  | | | | | |
| Extension Strength Deficit  at 12 months | ^1^ Depression yes/no |  | -2.076 | -18.2, 14.0 | 0.799 |
|  | ^2^ Depression yes/no, baseline confounders^a^ | BMI | -2.263 | -18.6, 14.1 | 0.785 |
|  | ^2^Depression yes/no, baseline confounders^a^, baseline score | BMI, baseline function | -2.756 | -18.9, 13.4 | 0.737 |
| ^1^ Univariate regression analysis  ^2^ Multivariate regression analysis  ^a^ Baseline confounders include Age, Gender, ASA I/II, BMI, and Surgical approach. | | | | | |

| **Supplemental Table 5.** Regression models for Extension Strength & *PC* with adjustment for baseline confounders and baseline Extension Strength scores. | | | | | |
| --- | --- | --- | --- | --- | --- |
| **Outcome** | **Predictor** | **sign** | **Beta** | **95%-CI** | ***P-*value** |
| Extension Strength  at 6 months | ^1^ PC yes/no |  | -4.371 | -16.4, 7.7 | 0.475 |
|  | ^2^ PC yes/no, baseline confounders^a^ | Age, sex, BMI | -3.791 | -14.6, 7.1 | 0.492 |
|  | ^2^ PC yes/no, baseline confounders^a^, baseline score | Sex, BMI, baseline function | 1.507 | -11.2, 8.2 | 0.759 |
|  | | | | | |
| Extension Strength  at 12 months | ^1^ PC yes/no |  | -8.895 | -21.9, 4.1 | 0.180 |
|  | ^2^ PC yes/no, baseline confounders^a^ | Age, sex, BMI | -9.280 | -20.1, 1.5 | 0.092 |
|  | ^2^ PC yes/no, baseline confounders^a^, baseline score | Age, sex, BMI, baseline function | -7.055 | -16.8, 2.7 | 0.157 |
| ^1^ Univariate regression analysis  ^2^ Multivariate regression analysis  ^a^ Baseline confounders include Age, Gender, ASA I/II, BMI, and Surgical approach.  PC, Pain Catastrophizing | | | | | |

| **Supplemental Table 6.** Regression models for Extension Strength Deficits of the affected leg compared to the non-affected leg & *PC* with adjustment for baseline confounders and baseline Extension Strength Deficit scores. | | | | | |
| --- | --- | --- | --- | --- | --- |
| **Outcome** | **Predictor** | **sign** | **Beta** | **95%-CI** | ***P-*value** |
| Extension Strength Deficit  at 6 months | ^1^ PC yes/no |  | 10.312 | -6.009, 26.6 | 0.214 |
|  | ^2^ PC yes/no, baseline confounders^a^ | Age | 2.838 | -14.2, 19.8 | 0.742 |
|  | ^2^ PC yes/no, baseline confounders^a^, baseline score | Age, baseline function | 1.997 | -14.8, 18.8 | 0.815 |
|  | | | | | |
| Extension Strength Deficit  at 12 months | ^1^ PC yes/no |  | 8.222 | -11.0, 27.5 | 0.400 |
|  | ^2^ PC yes/no, baseline confounders^a^ | BMI | 4.348 | -15.6, 24.3 | 0.667 |
|  | ^2^ PC yes/no, baseline confounders^a^, baseline score | BMI, baseline function | 3.456 | -16.5, 23.4 | 0.734 |
| ^1^ Univariate regression analysis  ^2^ Multivariate regression analysis  ^a^ Baseline confounders include Age, Gender, ASA I/II, BMI, and Surgical approach.  PC, Pain Catastrophizing | | | | | |

| **Supplemental Table 7.** Regression models for Extension Strength Endurance (SE) & *Anxiety* with adjustment for baseline confounders and baseline Extension SE scores. | | | | | |
| --- | --- | --- | --- | --- | --- |
| **Outcome** | **Predictor** | **Sign** | **Beta** | **95%-CI** | ***P-*value** |
| Extension SE  at 6 months | ^1^ Anxiety yes/no |  | -2.773 | -9.8, 4.3 | 0.438 |
|  | ^2^ Anxiety yes/no, baseline confounders^a^ | Age, Sex, BMI | -1.904 | -8.0, 4.2 | 0.536 |
|  | ^2^ Anxiety yes/no, baseline confounders^a^, baseline score | Sex, BMI, baseline function | 2.009 | -2.9, 6.9 | 0.419 |
|  | | | | | |
| Extension SE  at 12 months | ^1^ Anxiety yes/no |  | -4.601 | -11.8, 2.6 | 0.208 |
|  | ^2^ Anxiety yes/no, baseline confounders^a^ | Age, Sex, ASA, BMI | -2.796 | -8.6, 3.0 | 0.342 |
|  | ^2^ Anxiety yes/no, baseline confounders^a^, baseline score | Age, Sex, BMI, baseline function | 0.185 | -4.7, 5.0 | 0.940 |
| ^1^ Univariate regression analysis  ^2^ Multivariate regression analysis  ^a^ Baseline confounders include Age, Gender, ASA I/II, BMI, and Surgical approach. | | | | | |

| **Supplemental Table 8.** Regression models for Extension Strength Endurance (SE) Deficits of the affected leg compared to the non-affected leg & *Anxiety* with adjustment for baseline confounders and baseline Extension SE Deficit scores. | | | | | |
| --- | --- | --- | --- | --- | --- |
| **Outcome** | **Predictor** | **Sign** | **Beta** | **95%-CI** | ***P-*value** |
| Extension SE Deficit  at 6 months | ^1^ Anxiety yes/no |  | 3.472 | -5.4, 12.3 | 0.439 |
|  | ^2^ Anxiety yes/no, baseline confounders^a^ | Age | 2.591 | -6.4, 11.6 | 0.571 |
|  | ^2^ Anxiety yes/no, baseline confounders^a^, baseline score | Age, baseline function | -2.412 | -10.4, 5.6 | 0.552 |
|  | | | | | |
| Extension SE Deficit  at 12 months | ^1^ Anxiety yes/no |  | 3.763 | -14.3, 19.8 | 0.750 |
|  | ^2^ Anxiety yes/no, baseline confounders^a^ | - | 3.308 | -14.2, 20.8 | 0.710 |
|  | ^2^ Anxiety yes/no, baseline confounders^a^, baseline score | Baseline function | 1.833 | -16.0, 19.7 | 0.840 |
| ^1^ Univariate regression analysis  ^2^ Multivariate regression analysis  ^a^ Baseline confounders include Age, Gender, ASA I/II, BMI, and Surgical approach. | | | | | |

| **Supplemental Table 9.** Regression models for Extension Strength Endurance (SE) & *Depression* with adjustment for baseline confounders and baseline Extension SE score. | | | | | |
| --- | --- | --- | --- | --- | --- |
| **Outcome** | **Predictor** | **sign** | **Beta** | **95%-CI** | ***P-*value** |
| Extension SE  at 6 months | ^1^ Depression yes/no |  | -6.101 | -13.7, 1.5 | 0.117 |
|  | ^2^ Depression yes/no, baseline confounders^a^ | Age, Sex, BMI | -5.434 | -12.0, 1.1 | 0.103 |
|  | ^2^ Depression yes/no, baseline confounders^a^, baseline score | Sex, BMI, baseline function | -0.489 | -5.8, 4.8 | 0.856 |
|  | | | | | |
| Extension SE  at 12 months | ^1^ Depression yes/no |  | -7.181 | -15.0, 0.6 | 0.070 |
|  | ^2^ Depression yes/no, baseline confounders^a^ | Age, Sex, BMI | -6.771 | -13.0, -0.5 | 0.034 |
|  | ^2^Depression yes/no, baseline confounders^a^, baseline score | Age, Sex, BMI, baseline function | -1.585 | -6.8, 3.7 | 0.552 |
| ^1^ Univariate regression analysis  ^2^ Multivariate regression analysis  ^a^ Baseline confounders include Age, Gender, ASA I/II, BMI, and Surgical approach. | | | | | |

| **Supplemental Table 10.** Regression models for Extension Strength Endurance (SE) Deficits of the affected leg compared to the non-affected leg & *Depression* with adjustment for baseline confounders and baseline Extension SE Deficit score. | | | | | |
| --- | --- | --- | --- | --- | --- |
| **Outcome** | **Predictor** |  | **Beta** | **95%-CI** | ***P-*value** |
| Extension SE Deficit  at 6 months | ^1^ Depression yes/no |  | 2.791 | -6.8, 12.4 | 0.567 |
|  | ^2^ Depression yes/no, baseline confounders^a^ | Age | 1.569 | -8.2, 11.4 | 0.752 |
|  | ^2^ Depression yes/no, baseline confounders^a^, baseline score | Age, baseline function | -1.339 | -9.9, 7.2 | 0.758 |
|  | | | | | |
| Extension SE Deficit  at 12 months | ^1^ Depression yes/no |  | -4.825 | -23.2, 13.6 | 0.606 |
|  | ^2^ Depression yes/no, baseline confounders^a^ | - | -5.356 | -24.3, 13.5 | 0.577 |
|  | ^2^Depression yes/no, baseline confounders^a^, baseline score | Baseline function | -6.720 | -25.6, 12.2 | 0.485 |
| ^1^ Univariate regression analysis  ^2^ Multivariate regression analysis  ^a^ Baseline confounders include Age, Gender, ASA I/II, BMI, and Surgical approach. | | | | | |

| **Supplemental Table 11.** Regression models for Extension Strength Endurance (SE) & *PC* with adjustment for baseline confounders and baseline Extension SE scores. | | | | | |
| --- | --- | --- | --- | --- | --- |
| **Outcome** | **Predictor** | **sign** | **Beta** | **95%-CI** | ***P-*value** |
| Extension SE  at 6 months | ^1^ PC yes/no |  | -3.740 | -12.4, 4.9 | 0.394 |
|  | ^2^ PC yes/no, baseline confounders^a^ | Age, Sex, BMI | -3.556 | -11.2, 4.1 | 0.361 |
|  | ^2^ PC yes/no, baseline confounders^a^, baseline score | Sex, BMI, baseline function | -4.62 | -6.6, 5.7 | 0.882 |
|  | | | | | |
| Extension SE  at 12 months | ^1^ PC yes/no |  | -5.061 | -14.2, 4.0 | 0.274 |
|  | ^2^ PC yes/no, baseline confounders^a^ | Age, Sex, ASA, BMI | -5.741 | -13.2, 1.8 | 0.132 |
|  | ^2^ PC yes/no, baseline confounders^a^, baseline score | Age, Sex, BMI, baseline function | -1.891 | -8.2, 4.4 | 0.556 |
| ^1^ Univariate regression analysis  ^2^ Multivariate regression analysis  ^a^ Baseline confounders include Age, Gender, ASA I/II, BMI, and Surgical approach.  PC, Pain Catastrophizing | | | | | |

| **Supplemental Table 12.** Regression models for Extension Strength Endurance (SE) Deficits of the affected leg compared to the non-affected leg & *PC* with adjustment for baseline confounders and baseline Extension SE Deficit scores. | | | | | |
| --- | --- | --- | --- | --- | --- |
| **Outcome** | **Predictor** | **sign** | **Beta** | **95%-CI** | ***P-*value** |
| Extension SE Deficit  at 6 months | ^1^ PC yes/no |  | 3.997 | -6.8, 14.7 | 0.467 |
|  | ^2^ PC yes/no, baseline confounders^a^ | Age | 0.663 | -10.6, 11.9 | 0.907 |
|  | ^2^ PC yes/no, baseline confounders^a^, baseline score | Age, baseline function | -2.057 | -11.9, 7.8 | 0.680 |
|  | | | | | |
| Extension SE Deficit  at 12 months | ^1^ PC yes/no |  | 6.419 | -15.6, 28.4 | 0.566 |
|  | ^2^ PC yes/no, baseline confounders^a^ | - | 4.893 | -18.1, 27.9 | 0.676 |
|  | ^2^ PC yes/no, baseline confounders^a^, baseline score | Baseline function | 0.973 | -22.7, 24.7 | 0.936 |
| ^1^ Univariate regression analysis  ^2^ Multivariate regression analysis  ^a^ Baseline confounders include Age, Gender, ASA I/II, BMI, and Surgical approach.  PC, Pain Catastrophizing | | | | | |

| **Supplemental Table 13.** Regression models for Extension ROM & *Anxiety* with adjustment for baseline confounders and baseline Extension ROM scores. | | | | | |
| --- | --- | --- | --- | --- | --- |
| **Outcome** | **Predictor** | **sign** | **Beta** | **95%-CI** | ***P-*value** |
| Extension ROM  at 6 months | ^1^ Anxiety yes/no |  | -0.6336 | -2.6, 1.4 | 0.533 |
|  | ^2^ Anxiety yes/no, baseline confounders^a^ | Approach | -0.964 | -3.0, 1.1 | 0.349 |
|  | ^2^ Anxiety yes/no, baseline confounders^a^, baseline score | Approach, baseline function | -0.970 | -3.0, 1.0 | 0.342 |
|  | | | | | |
| Extension ROM  at 12 months | ^1^ Anxiety yes/no |  | -0.318 | -1.7, 1.1 | 0.649 |
|  | ^2^ Anxiety yes/no, baseline confounders^a^ | Sex, approach | -0.314 | -1.7, 1.1 | 0.654 |
|  | ^2^ Anxiety yes/no, baseline confounders^a^, baseline score | Sex, approach, baseline function | -0.434 | -1.8, 1.0 | 0.537 |
| ^1^ Univariate regression analysis  ^2^ Multivariate regression analysis  ^a^ Baseline confounders include Age, Gender, ASA I/II, BMI, and Surgical approach. | | | | | |

| **Supplemental Table 14.** Regression models for Extension ROM & *Depression* with adjustment for baseline confounders and baseline Extension ROM score. | | | | | |
| --- | --- | --- | --- | --- | --- |
| **Outcome** | **Predictor** | **sign** | **Beta** | **95%-CI** | ***P-*value** |
| Extension ROM  at 6 months | ^1^ Depression yes/no |  | -2.136 | -4.3, 0.0 | 0.053 |
|  | ^2^ Depression yes/no, baseline confounders^a^ | Approach | -2.759 | -4.9, -0.6 | 0.013 |
|  | ^2^ Depression yes/no, baseline confounders^a^, baseline score | Approach, baseline function | -2.854 | -5.0, -0.7 | 0.009 |
|  | | | | | |
| Extension ROM  at 12 months | ^1^ Depression yes/no |  | -0.882 | -2.4, 0.6 | 0.241 |
|  | ^2^ Depression yes/no, baseline confounders^a^ | Sex, approach | -0.889 | -2.4, 0.6 | 0.240 |
|  | ^2^Depression yes/no, baseline confounders^a^, baseline score | Sex, approach, baseline function | -0.959 | -2.4, 0.5 | 0.200 |
| ^1^ Univariate regression analysis  ^2^ Multivariate regression analysis  ^a^ Baseline confounders include Age, Gender, ASA I/II, BMI, and Surgical approach. | | | | | |

| **Supplemental Table 15.** Regression models for Extension ROM & *PC* with adjustment for baseline confounders and baseline Extension ROM scores. | | | | | |
| --- | --- | --- | --- | --- | --- |
| **Outcome** | **Predictor** | **sign** | **Beta** | **95%-CI** | ***P-*value** |
| Extension ROM  at 6 months | ^1^ PC yes/no |  | -0.415 | -2.8, 2.0 | 0.733 |
|  | ^2^ PC yes/no, baseline confounders^a^ | Approach | -0.736 | -3.2, 1.7 | 0.558 |
|  | ^2^ PC yes/no, baseline confounders^a^, baseline score | Approach, baseline function | -0.884 | -3.3, 1.5 | 0.474 |
|  | | | | | |
| Extension ROM  at 12 months | ^1^ PC yes/no |  | 0.163 | -1.5, 1.8 | 0.846 |
|  | ^2^ PC yes/no, baseline confounders^a^ | Sex, approach | 0.660 | -1.0, 2.3 | 0.439 |
|  | ^2^ PC yes/no, baseline confounders^a^, baseline score | Sex, approach, baseline function | 0.688 | -1.0, 2.4 | 0.415 |
| ^1^ Univariate regression analysis  ^2^ Multivariate regression analysis  ^a^ Baseline confounders include Age, Gender, ASA I/II, BMI, and Surgical approach.  PC, Pain Catastrophizing | | | | | |

| **Supplemental Table 16.** Regression models for TUG & *Anxiety* with adjustment for baseline confounders and baseline TUG score. | | | | | |
| --- | --- | --- | --- | --- | --- |
| **Outcome** | **Predictor** | **Sign** | **Beta** | **95%-CI** | ***P-*value** |
| TUG at 6 months | ^1^ Anxiety yes/no |  | -0.372 | -1.3, 0.6 | 0.450 |
|  | ^2^ Anxiety yes/no, baseline confounders^a^ | Age, BMI | -0.187 | -1.1, 0.7 | 0.685 |
|  | ^2^ Anxiety yes/no, baseline confounders^a^, baseline score | Age, BMI, baseline function | -0.236 | -1.0, 0.6 | 0.552 |
|  | | | | | |
| TUG at 12 months | ^1^ Anxiety yes/no |  | -0.178 | -1.2, 0.8 | 0.720 |
|  | ^2^ Anxiety yes/no, baseline confounders^a^ | Age, ASA | -0.065 | -1.0, 0.8 | 0.888 |
|  | ^2^ Anxiety yes/no, baseline confounders^a^, baseline score | Age, baseline function | -0.623 | -1.0, 0.6 | 0.623 |
| ^1^ Univariate regression analysis  ^2^ Multivariate regression analysis  ^a^ Baseline confounders include Age, Gender, ASA I/II, BMI, and Surgical approach. | | | | | |

| **Supplemental Table 17.** Regression models for TUG & *Depression* with adjustment for baseline confounders and baseline TUG score. | | | | | |
| --- | --- | --- | --- | --- | --- |
| **Outcome** | **Predictor** | **sign** | **Beta** | **95%-CI** | ***P-*value** |
| TUG at 6 months | ^1^ Depression yes/no |  | 0.258 | -0.8, 1.3 | 0.630 |
|  | ^2^ Depression yes/no, baseline confounders^a^ | Age, BMI | 0.441 | -0.5, 1.4 | 0.377 |
|  | ^2^ Depression yes/no, baseline confounders^a^, baseline score | Age, BMI, baseline function | -0.124 | -1.0, 0.7 | 0.774 |
|  | | | | | |
| TUG at 12 months | ^1^ Depression yes/no |  | 0.981 | -0.1, 2.0 | 0.066 |
|  | ^2^ Depression yes/no, baseline confounders^a^ | Age, ASA | 1.137 | 0.2, 2.1 | 0.020 |
|  | ^2^Depression yes/no, baseline confounders^a^, baseline score | Age, baseline function | 0.494 | -0.3, 1.3 | 0.246 |
| ^1^ Univariate regression analysis  ^2^ Multivariate regression analysis  ^a^ Baseline confounders include Age, Gender, ASA I/II, BMI, and Surgical approach. | | | | | |

| **Supplemental Table 18.** Regression models for TUG & *PC* with adjustment for baseline confounders and baseline TUG scores. | | | | | |
| --- | --- | --- | --- | --- | --- |
| **Outcome** | **Predictor** | **sign** | **Beta** | **95%-CI** | ***P-*value** |
| TUG at 6 months | ^1^ PC yes/no |  | 0.036 | -1.1, 1.2 | 0.951 |
|  | ^2^ PC yes/no, baseline confounders^a^ | Age, BMI | 0.911 | -0.2, 2.0 | 0.104 |
|  | ^2^ PC yes/no, baseline confounders^a^, baseline score | Age, BMI, baseline function | 0.337 | -0.6, 1.3 | 0.486 |
|  | | | | | |
| TUG at 12 months | ^1^ PC yes/no |  | -0.103 | -1.2, 1.1 | 0.863 |
|  | ^2^ PC yes/no, baseline confounders^a^ | Age, ASA | 0.622 | -0.5, 1.7 | 0.263 |
|  | ^2^ PC yes/no, baseline confounders^a^, baseline score | Age, baseline function | -0.360 | -1.3, 0.6 | 0.458 |
| ^1^ Univariate regression analysis  ^2^ Multivariate regression analysis  ^a^ Baseline confounders include Age, Gender, ASA I/II, BMI, and Surgical approach.  PC, Pain Catastrophizing | | | | | |
